# Additive Manufacturing of Aerospace Composites: A Critical Review of the Material–Process–Design Interplay and Prospects for Application

**DOI:** 10.3390/ma18184280

**Published:** 2025-09-12

**Authors:** Chenwei Shen, Yanbing Guo, Zhikang Shen, Fei Yan, Ning Zhong

**Affiliations:** 1 Institute of Marine Materials Science and Engineering, College of Ocean Science and Engineering, Shanghai Maritime University, Shanghai 201306, China; 202210415039@stu.shmtu.edu.cn (C.S.); ningzhong@shmtu.edu.cn (N.Z.); 2 College of Engineering and Technology, Southwest University, Chongqing 400715, China; zhikangshen@swu.edu.cn; 3 School of Automobile Engineering, Wuhan University of Technology, Wuhan 430070, China; fyan001@whut.edu.cn

**Keywords:** composite additive manufacturing, aerospace composites, critical review, additive manufacturing design optimization, materials-processes-properties relationship, technical path

## Abstract

In aeronautical applications, composite additive manufacturing (CAM) is transforming aircraft design by enabling unprecedented lightweighting and functional integration. However, industrial adoption remains limited due to insufficient understanding of the complex interplay among materials, processes, designs, and performance. Existing reviews lack an integrated analytical framework; thus, this study pioneers a closed-loop “Material-Process-Design-Performance (MPDP)” framework for critical analysis. We systematically categorize aerospace-relevant composite systems and core AM technologies, emphasizing their interactions and constraints. Crucially, we demonstrate how AM design bridges material limitations and performance breakthroughs. Validation via aerospace case studies confirms the framework’s efficacy and reveals core bottlenecks: performance consistency, quality control, and certification gaps. We argue that transitioning from isolated optimization to collaborative co-optimization of materials, processes, and design is essential for CAM’s advancement. Finally, we propose a strategic development pathway to guide future research and industrial translation, particularly for next-generation aircraft and spacecraft systems.

## 1. Introduction

For aeronautical structures, the demand for weight reduction directly translates to fuel savings and reduced carbon emissions over an aircraft’s lifecycle. The pursuit of weight reduction, fuel efficiency, and superior structural integrity continually drives aerospace innovation [1]. High-performance, lightweight composite materials have emerged as a cornerstone solution, valued for their exceptional specific strength, specific stiffness, tailorable properties, and corrosion resistance. Consequently, their adoption has expanded significantly across aerospace structures, from primary load-bearing components to interior elements [2,3].

However, traditional composite manufacturing (e.g., hand layup, RTM, AFP) is limited by long cycle times, high costs, geometric constraints, and material waste [4,5]. AM, or 3D printing, presents a transformative alternative, offering unprecedented freedom in design complexity, significant waste reduction, and accelerated prototyping and production [6]. The convergence of AM with composite materials (CAM) promises to revolutionize aerospace structural design, functional integration, and performance customization [7,8]. Proponents highlight CAM’s potential for drastic weight savings via topology optimization and its inherent material efficiency, which could substantially lower carbon emissions across a component’s lifecycle [9]. Market projections underscore this optimism, forecasting the global aerospace CAM market to surge from approximately 829 million in 2023 to over 3.6 billion by 2032, reflecting a robust compound annual growth rate (CAGR) of 20.24% [10].

Significant progress validates this potential. Leading aerospace entities (e.g., Boeing, Airbus, GE Aviation) have successfully deployed CAM for structural and non-structural parts, demonstrating tangible benefits in weight reduction and performance enhancement [3,11,12]. Notable examples include complex, topology-optimized wing ribs with internal lattice structures achieving significant material savings [3,13], and exploration into intricate engine components aiming for cost reduction and efficiency gains [12].

Despite these advancements and considerable enthusiasm, the path to large-scale, certified aerospace application of CAM is fraught with significant, interconnected challenges that expose critical controversies and diverging viewpoints within the research community:

1. Performance Determinism and Consistency: A key debate in achieving reliable performance revolves around whether to prioritize material innovation or control over process-induced microstructures. Challenges like fiber orientation, distribution, and defects (e.g., warping, delamination) lead to variability in part quality. The research community is divided on whether defect mitigation or advancements in process control and in situ monitoring are essential for achieving consistency.
2. Economic Viability and Scalability: There is significant disagreement about the scalability and cost-effectiveness of current CAM technologies, particularly for large structures. While CAM is advantageous for complex prototypes, its competitiveness with established high-throughput methods for mass production is debated due to issues with production speed and cost. Researchers are split on focusing on automation versus radical innovations to increase deposition rates.
3. Certification and Standardization Lag: A major bottleneck is the lack of established certification pathways for CAM, especially in the highly regulated aerospace sector. There is controversy over whether existing aerospace certification frameworks are sufficient or if new, CAM-specific standards are necessary for material specs, process qualification, and non-destructive evaluation. This lack of standards hampers the transition of CAM parts into certified flight applications.

The aerospace CAM research landscape is marked by “dynamic progress tempered by profound systemic challenges.” While there are successes and recognized potential, the field faces unresolved scientific questions regarding process–structure–property relationships, technological barriers to scaling, and a lack of regulatory frameworks. These challenges result in slower innovation, high costs for complex components, and limited performance improvements, confining CAM’s application primarily to non-critical structures, prototypes, and tooling.

Previous reviews often addressed CAM aspects in isolation, overlooking interdependencies. This review introduces a closed-loop “Material-Process-Design-Performance” (MPDP) framework to analyze the aerospace CAM domain. It explores material–process compatibility, highlights how design innovation drives performance, and uses feedback to guide material and process choices. By integrating state-of-the-art applications with core challenges, the review suggests strategic pathways for future research to advance CAM in aerospace.

To ensure a thorough analysis, a systematic literature search was conducted across key databases like Web of Science and Scopus, focusing on “additive manufacturing/3D printing” and “aerospace composites,” with particular emphasis on literature published between 2010 and 2025.

## 2. Key Properties Required for Aerospace Applications

### 2.1. High Strength and Stiffness

Aeronautical components (e.g., wing spars, landing gear) must withstand cyclic loads and fatigue, necessitating CAM designs with topology-optimized stress paths. Materials must simultaneously possess high elastic modulus and yield strength, achieving a high ratio relative to their density, to endure aerodynamic loads and inertial forces. AM’s design freedom enables advanced methodologies like topology optimization (TO) and lattice structures, which are impossible with traditional manufacturing. This enables the achievement of maximum lightweighting while meeting or even exceeding stiffness and strength requirements. Particularly for CAM, its capability to achieve continuous fiber placement along primary stress paths allows it to fully harness the anisotropic advantages of the material. This represents a capability unmatched by traditional metal processing or isotropic AM techniques [14]. For instance, Airbus utilized TO and AM to produce an A350 cabin bracket connector from titanium alloy Ti-6Al-4V, achieving significant weight reduction while maintaining high strength [15].

### 2.2. Thermal Resistance

Aerospace vehicles encounter extreme thermal conditions during operation, such as exposure to high-temperature combustion gases and aerodynamic heating. Materials are required to maintain stable performance at elevated temperatures, exhibiting well-controlled coefficients of thermal expansion (CTE), oxidation resistance, and resistance to hot corrosion. AM enhances the thermal resistance of composites in ways traditional methods cannot. Its core advantage lies not merely in processing high-temperature-resistant materials, but in the capability to fabricate intricate composite structures with optimized thermal management functionality.

For instance, in the case of ceramic matrix composites (CMCs) and metal matrix composites (MMCs), CAM enables the production of monolithic components featuring complex internal cooling channels—such as turbine blades and combustor liners—which are unattainable through conventional manufacturing techniques [5,13]. This sophisticated internal channel design significantly enhances heat exchange efficiency, thereby allowing components to operate safely at substantially higher temperatures.

### 2.3. Lightweighting

Reducing structural weight is a paramount objective in the aerospace sector, as weight increases lead to higher fuel consumption and diminished flight efficiency. CAM elevates the potential for lightweighting to unprecedented levels. It not only inherits AM’s advantages in structural TO but also achieves synergistic material–structure optimization by integrating lightweight, high-strength fibers (e.g., carbon fiber) with high-performance polymer matrices.

The core advantage of CAM lies in its capability for “load-directed fiber reinforcement”. This enables the precise placement of reinforcing fibers in regions and directions experiencing the highest loads, while retaining lightweight matrix structures in less critical areas. This approach effectively eliminates the material redundancy commonly found in traditional manufacturing [16].

A highly representative case is the exploration by the Commercial Aircraft Corporation of China (COMAC) for large primary load-bearing structures within its C929 wide-body airliner program. Utilizing continuous carbon fiber-reinforced poly(aryl ether ketone) (PAEK) composite, they successfully prototyped an 8.2 m long wing spar structure via AM technology. This work validated CAM’s feasibility for fabricating large, complex monolithic composites and demonstrated its potential for lightweighting primary structures beyond the capabilities of metallic AM [17].

Consequently, the lightweighting achieved through CAM represents a profound exploitation of material properties and structural efficiency, fundamentally constrained by the imperative to ensure sufficient stiffness and strength to prevent buckling or resonance phenomena.

### 2.4. Corrosion Resistance and Aging Resistance

Aerospace components are required to operate long-term in diverse environments, necessitating materials with excellent corrosion resistance and aging resistance to withstand the degradation caused by chemical media and environmental factors. Confronting the challenges of corrosion and aging, AM offers innovative solutions that transcend traditional approaches such as surface coatings or monolithic corrosion-resistant alloys. Its core advantage lies in the capability to fabricate functionally graded materials (FGMs) [18]. For instance, it enables the creation of a seamless transition to corrosion-resistant element-enriched alloy compositions at the component surface, or the graded distribution of shielding nano-fillers (e.g., graphene) within a composite matrix. This results in a monolithic, integrally bonded protective layer devoid of distinct interfaces [19]. Furthermore, by leveraging AM’s precise shape control capability, specific microstructures possessing intrinsic aging or erosion resistance (e.g., bio-inspired porous architectures) can be directly fabricated within critical regions such as fluid-wetted surfaces or stress concentration zones. This facilitates a paradigm shift from passive protection to active design integration [20].

## 3. Fundamentals of CAM for Aerospace

### 3.1. Aerospace-Relevant Composite Materials for AM

#### 3.1.1. Thermoplastic Composites

In aerospace AM, composites formed from thermoplastic matrices such as Polyether Ether Ketone (PEEK), Polyether Ketone Ketone (PEKK), and Polyetherimide (ULTEM) reinforced with carbon/glass fibers have emerged as key materials for metal replacement. This is driven by their lightweight nature, high specific strength, and resistance to extreme environments (PEEK/PEKK withstand >250 °C, ULTEM offers inherent flame retardancy) [21]. Regarding weight reduction and performance enhancement, carbon fiber-reinforced PEEK components can achieve 40–60% weight savings compared to traditional aluminum alloys, making them highly attractive for structures such as brackets and secondary load-bearing beams [22]. For aircraft interiors, FDM-processed ULTEM/PEEK meets FAA flammability and low-smoke toxicity requirements, enabling rapid certification of cabin components. Thermoplastics offer superior impact toughness and chemical resistance compared to epoxy resins, making them ideal for fuel system components. Their recyclability and AM’s near-net-shape capability also promote sustainable manufacturing of complex structures.

However, thermoplastic composites have significant limitations for primary load-bearing structures, most notably poor interlaminar (Z-direction) strength, which is often only 30–50% of the in-plane strength. This issue of process-induced anisotropy is discussed further in Section 6.1.2 [23] as it severely restricts their application in primary load-bearing structures. At the process level, the high printing temperatures (380–450 °C) and reliance on temperature-controlled build chambers significantly increase equipment costs and energy consumption [23]. Concurrently, unified airworthiness certification standards are lacking within the industry, and the surface accuracy of printed parts often requires additional post-processing to meet assembly tolerances.

The interplay between material properties and processing parameters is key to understanding these application bottlenecks. For instance, in FDM processes, the high viscosity of continuous fiber-reinforced thermoplastic melts is a primary cause of elevated porosity and insufficient interlayer bonding during printing [24]. In contrast, direct ink writing (DIW) technology reduces apparent viscosity (<100 Pa·s) through solvent-assisted or photocurable prepolymers, theoretically enabling denser structures [25]. However, DIW processes rely on time-consuming post-curing or solvent removal steps, negatively impacting production efficiency.

The current focus of thermoplastic composite AM is on improving interlaminar performance and reducing porosity through advancements in continuous fiber reinforcement and in situ consolidation. Its core value is in balancing “lightweighting-environmental resistance-design freedom.” In the short term, it has applications in secondary structures and interior components, while long-term use in primary structures requires improvements in process efficiency and strong certification frameworks.

#### 3.1.2. Thermoset Composites

Carbon fiber-reinforced thermoset composites based on epoxy and polyimide matrices exhibit unique value in the aerospace sector due to their high-precision forming potential and exceptional temperature resistance. However, their adaptability to AM faces significant challenges. At the process level, vat photopolymerization-based AM techniques (e.g., stereolithography, SLA/digital light processing, DLP) can fabricate complex thin-walled components (such as sensor housings) with precision down to ~50 μm. Nevertheless, achieving high-content, uniformly dispersed fiber reinforcement within the resin matrix remains a significant technical hurdle [6]. In contrast, material extrusion processes (e.g., DIW) can directly process prepreg fiber bundles, theoretically yielding superior interlaminar strength compared to thermoplastics. However, these processes suffer from slow printing speeds, and large-scale components are prone to warping due to uneven shrinkage during curing.

The core bottleneck in additive manufacturing (AM) is the curing process. Epoxy resins require extended thermal curing at 80–120 °C, while polyimides need even higher temperatures, often exceeding 300 °C. This high-temperature post-processing leads to significant energy consumption and induces residual stresses. Additionally, the layer-by-layer deposition in AM results in incomplete crosslinking, causing Z-direction strength losses of 20–40% [26] and micro-porosity levels often exceeding 3%, which is above the aerospace standard of <1%. Future advancements depend on developing low-temperature, rapid-cure resin systems and hybrid strategies that combine AM with autoclave post-curing, aiming for a balance of “high precision,” “high temperature resistance,” and “mechanical performance” similar to traditional manufacturing.

#### 3.1.3. Metal Matrix Composites (MMCs)

**Figure 1:**
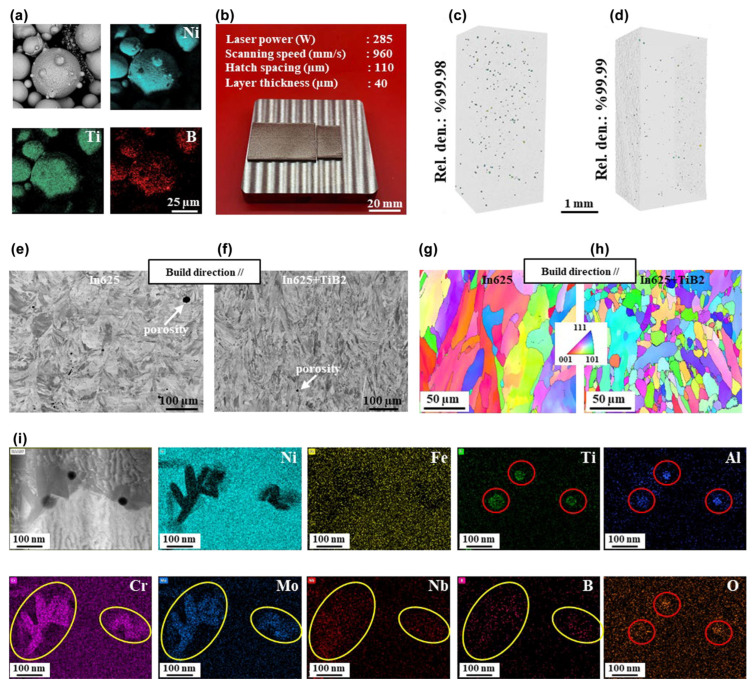
(**a**) SEM micrograph of TiB_2_-coated Inconel 625 (In625) powder after mixing; (**b**) In625+TiB_2_ sample fabricated via LPBF using an EOS M290 system (shown in as-built condition on substrate); (**c**,**d**) 3D CT reconstructions depicting porosity formation in In625 and In625+TiB_2_ samples during printing; (**e**,**f**) SEM images of In625 and In625+TiB_2_ samples; (**g**,**h**) EBSD orientation maps of In625 and In625+TiB_2_ (revealing grain refinement phenomena); (**i**) STEM/EDX elemental distribution map of In625+TiB_2_ (showing interdiffusion zones of Cr, Mo, Nb, Ti, and B elements) [27].

MMCs are typically composed of a metallic matrix (e.g., aluminum, titanium) and a reinforcing phase (e.g., SiC particles, carbon fibers). By integrating lightweight metals with high-hardness, high-modulus ceramic particles, MMCs aim to achieve a synergistic combination of lightweighting and extreme thermal/wear resistance. This grants them significant application potential for aero-engine hot-section components and high-stiffness structural parts [15]. However, when fabricating MMCs via AM, particularly high-temperature processes like laser powder bed fusion (LPBF), the physicochemical interactions between the constituent materials emerge as the core challenge. The intense, transient heat in AM processes like LPBF can promote deleterious interfacial reactions in MMCs, forming brittle intermetallic compounds (e.g., TiC, Ti_5_Si_3_) that weaken interfacial bonding and reduce toughness (see Section 6.1.1) [27]. Concurrently, the substantial coefficient of thermal expansion (CTE) mismatch between the reinforcement and the matrix generates significant residual stresses during rapid solidification, potentially inducing microcrack initiation [28]. In melt pool processing, complex convective flows may cause lower-density SiC particles to agglomerate, creating stress concentrations and leading to inhomogeneous properties in the component. Thus, while MMCs offer great performance potential, achieving this through AM technology requires precise control of the melt pool’s thermodynamic and kinetic processes to mitigate harmful interfacial reactions and ensure uniform reinforcement distribution. Figure 1 shows SEM and EDS microanalysis of a composite powder made of Inconel 625 (In625) and TiB_2_ particles, which maintain a spherical morphology, with effective surface modification observed on the In625 particles.

#### 3.1.4. Ceramic Matrix Composites (CMCs)

**Figure 2:**
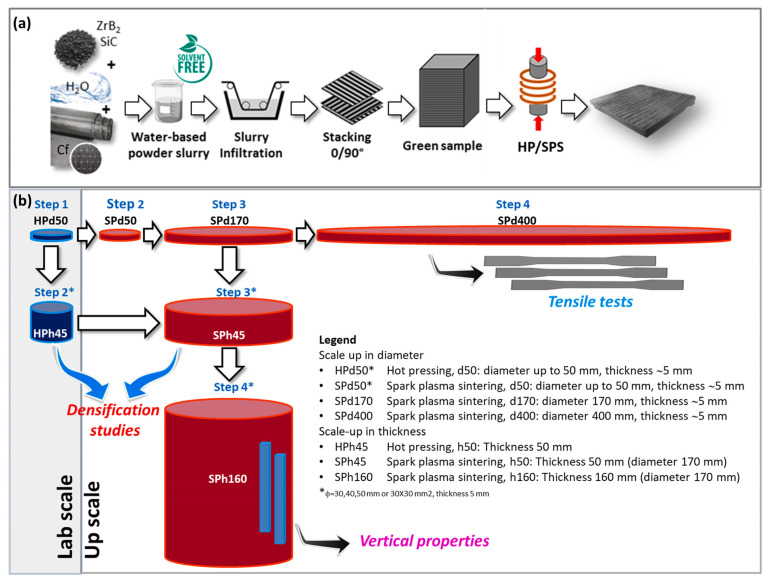
(**a**) Schematic illustration of the fabrication process for ultra-high-temperature ceramic matrix composites (UHTCMCs); (**b**) underpinning scientific principles for scaled production of UHTCMCs (continuous fiber-reinforced structures) [29].

CMCs, comprising a ceramic matrix (e.g., SiC) and reinforcing fibers (e.g., SiC fiber), represent a critical class of materials developed to withstand extreme temperatures exceeding 1300 °C [29]. Compared to traditional CMC manufacturing processes, which are often lengthy and costly (e.g., chemical vapor infiltration, CVI), AM offers a novel pathway for the low-cost and rapid fabrication of complex-shaped CMC components. For instance, vat photopolymerization techniques (e.g., DLP) enable the printing of slurries containing ceramic precursors (e.g., silicone-based resins) and inert ceramic fillers. This is followed by a high-temperature pyrolysis process converting the polymeric precursor into the ceramic matrix [30]. The primary advantage of this approach is its capability for single-step fabrication of net-shape or near-net-shape CMC components featuring intricate internal structures (e.g., cooling channels), thereby eliminating costly machining steps. However, significant challenges remain: the substantial volumetric shrinkage (20–30%) occurring during pyrolysis can cause component cracking and severely compromise dimensional accuracy. Mitigation strategies necessitate optimizing precursor chemistry and incorporating inert fillers [30]. Furthermore, the effective introduction and retention of continuous fiber integrity and orientation within this process—essential for achieving meaningful toughening—remains a key research focus and considerable challenge. Figure 2 illustrates the fabrication process for ultra-high-temperature ceramic matrix composites (UHTCMCs) (Figure 2a) and a scale-up strategy transitioning from laboratory to industrial production (Figure 2b).

### 3.2. Advanced AM Technologies for Composites

This section provides a critical assessment of mainstream CAM technologies from the perspective of aerospace applications. The analysis will focus on the advantages, limitations, and potential impacts of each technology on the performance of the final product when processing specific composite material systems.

#### 3.2.1. Fused Deposition Modeling (FDM)

**Figure 3:**
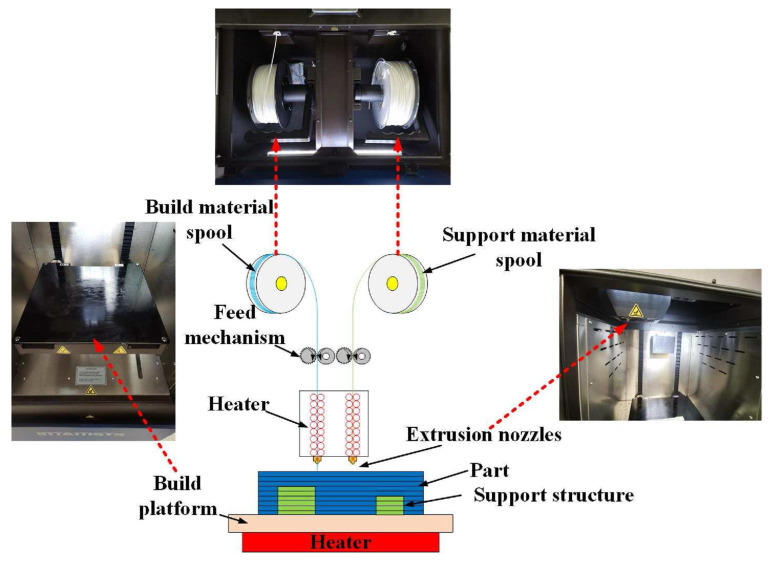
Schematic diagram of the FDM process principle [32].

FDM constructs parts layer-by-layer by melting and extruding thermoplastic filaments [24,31]. Its working principle is illustrated in Figure 3. Its primary advantages in the aerospace sector include relatively low equipment costs, high technological maturity, and the capability to process certified high-performance thermoplastics such as ULTEM 9085 and PEKK. These attributes make it highly suitable for rapid prototyping, tooling/jigs, and non-load-bearing interior components [22,32,33]. However, inherent limitations of FDM restrict its application in primary load-bearing structures. Firstly, interlayer bonding strength constitutes its primary weakness, resulting in significantly inferior mechanical properties along the build (Z) direction compared to the X-Y plane. Secondly, for continuous fiber-reinforced composites, the FDM process struggles to eliminate inter-filament voids completely, leading to elevated porosity in final parts that compromises overall performance. Furthermore, fabricating large or geometrically complex components necessitates the design and removal of substantial support structures, increasing manufacturing complexity and post-processing workload.

#### 3.2.2. Selective Laser Sintering (SLS)

**Figure 4:**
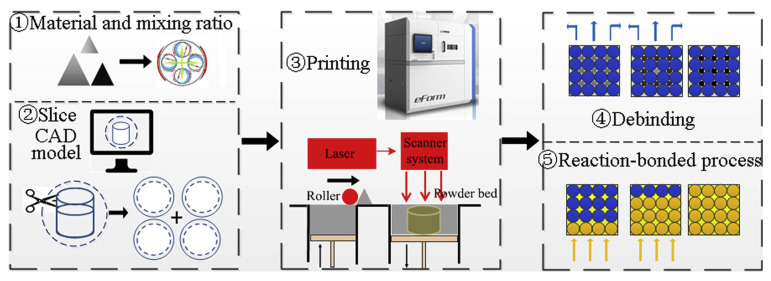
Schematic diagram of the SLS process principle [36].

SLS forms parts by selectively sintering material within a powder bed using a laser [34,35]. Its working principle is illustrated in Figure 4. SLS offers a unique advantage in fabricating polymer matrix composite components with complex internal geometries (e.g., ducts, lattices), as the unsintered powder acts as natural support, eliminating the need to design and remove dedicated support structures [36]. This capability makes SLS particularly well suited for producing intricate environmental control system ducts or monolithic UAV structural components. However, its limitations are equally significant: SLS parts typically exhibit high surface roughness (Ra > 20 μm), necessitating post-processing steps such as sanding and polishing to meet stringent aerospace surface finish requirements [36,37]. Furthermore, the range of high-performance composite powders suitable for SLS is limited, and the powder recycling/reuse process can lead to degradation of material properties, increasing complexity in quality control.

#### 3.2.3. Laser Metal Deposition (LMD)

**Figure 5:**
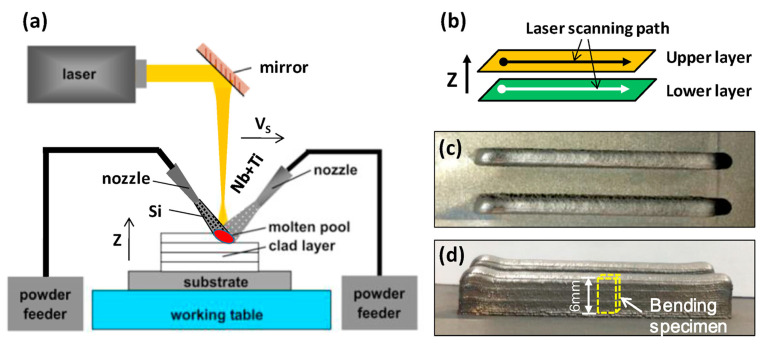
Schematic of dual-powder feeding in LMD (**a**), single-track scanning strategy (**b**), and top-view (**c**) and side-view (**d**) photographs of a deposited sample [38].

In the LMD process, a laser generates a molten pool on the substrate surface while metal or composite powder is directed into the pool, depositing material layer-by-layer as the laser traverses [38]. Its working principle is illustrated in Figure 5. The primary advantages of LMD are its high deposition efficiency and potential for manufacturing/repairing large-scale components. It is particularly well suited for surface hardening or damage repair of high-value parts such as aero-engine blades. The technology also enables the fabrication of FGMs, for instance, by depositing wear-resistant or corrosion-resistant composite layers onto a component’s surface. However, LMD exhibits relatively low geometric accuracy and surface finish, typically necessitating subsequent machining operations. Furthermore, the substantial heat input readily induces significant thermal stresses and distortion in thin-walled or complex geometries. This constitutes a critical challenge requiring resolution for the manufacturing of precision structural components.

#### 3.2.4. Selective Laser Melting (SLM)

**Figure 6:**
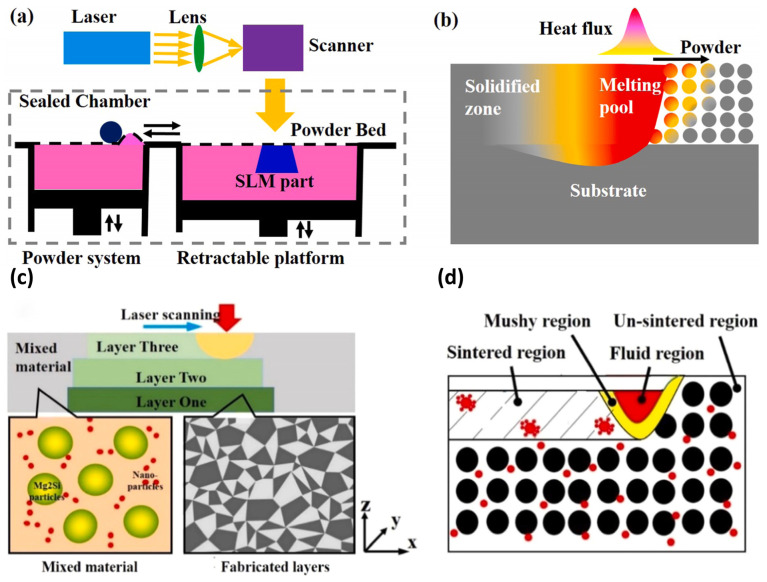
(**a**) Working principle of SLM; (**b**) SLM fabrication process of powder material; (**c**) SLM fabrication of multi-component thermoelectric powder embedded with nanoparticles; (**d**) four characteristic regions within the powder bed [39].

In the SLM process, a high-energy laser beam selectively melts metal or metal matrix composite (MMC) powder layer-by-layer within a powder bed [39]. Its working principle is illustrated in Figure 6. SLM is renowned for its high dimensional accuracy and capability to fabricate geometries of extreme complexity, making it an ideal choice for producing mission-critical aerospace components such as lightweight lattice structures and turbine blades with optimized cooling channels from metals and MMCs. However, the technology faces significant challenges: The rapid melt-solidification process generates substantial thermal stresses, inducing significant distortion or even cracking in components. Mitigation requires sophisticated process parameter optimization and post-build heat treatment [40]. Concurrently, the “balling effect” compromises surface quality, while unmelted powder particles can serve as potential sites for internal defects.

#### 3.2.5. Electron Beam Melting (EBM)

**Figure 7:**
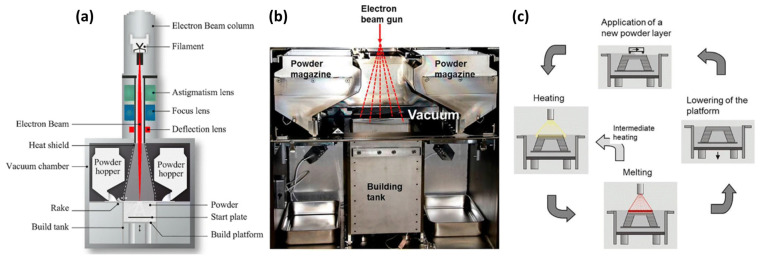
(**a**) Equipment details, (**b**) process chamber, and (**c**) layer formation in a typical EBM process [41].

EBM utilizes an electron beam in a vacuum environment to melt metallic powder. While predominantly employed for metal processing, it demonstrates unique potential for fabricating MMCs [41,42]. Its working principle is illustrated in Figure 7. Its core advantages include the following: Firstly, the high-vacuum environment (~10^−5^ mbar) effectively prevents oxidation of reactive metallic matrices (e.g., titanium, aluminum) and certain reinforcements at elevated temperatures, thereby ensuring material chemical purity and interfacial integrity. Secondly, EBM’s inherent capability for high-temperature preheating and inter-layer temperature maintenance (operating temperatures ≥1000 °C) significantly reduces thermal gradients during rapid melt solidification. This effectively mitigates substantial residual stresses arising from the CTE mismatch between matrix and reinforcement—a critical factor for suppressing cracking in MMCs [43]. However, EBM faces challenges in MMC fabrication: Potential interactions between the electron beam and non-conductive ceramic reinforcements (e.g., SiC) can cause electrostatic charge accumulation and beam deflection, compromising geometric accuracy. Concurrently, ensuring uniform reinforcement distribution within the powder bed and during melting, while preventing segregation induced by gravitational or electromagnetic forces, remains a critical challenge for achieving homogeneous properties in MMC components.

#### 3.2.6. Direct Ink Writing (DIW) for Fiber-Reinforced Composites

DIW is a process that constructs complex structures layer-by-layer through the precise extrusion and deposition of viscoelastic–plastic inks [25]. The technology’s foremost advantage lies in its exceptional material versatility, enabling processing of diverse composite systems—ranging from polymers to ceramics and short fibers to continuous fibers—while facilitating multi-material printing. This capability offers substantial potential for fabricating FGMs and integrated electronics [25,44].

**Figure 8:**
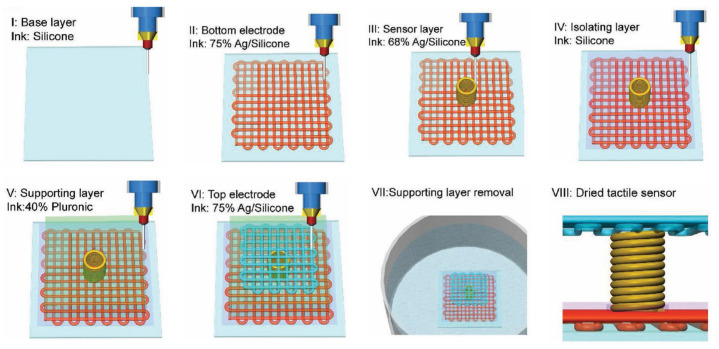
Printing of functional inks incorporating sensors using multi-material DIW [25].

Within aerospace, DIW currently operates at the spacecraft functional prototype stage. Its capacity for room temperature processing of continuous fiber–thermoset resin systems prevents thermal damage to reinforcements, achieving significantly superior fiber retention rates and lower porosity compared to FDM. DIW also enables multi-material co-printing, as demonstrated in Figure 8.

However, DIW exhibits relatively slow printing speeds, and the performance of final components is highly dependent on ink formulation and subsequent curing/sintering processes. This significantly increases process development complexity. Maintaining ink printability at high fiber loadings constitutes a critical challenge for advancing DIW toward structural load-bearing applications.

#### 3.2.7. Automated Fiber Placement (AFP)

**Figure 9:**
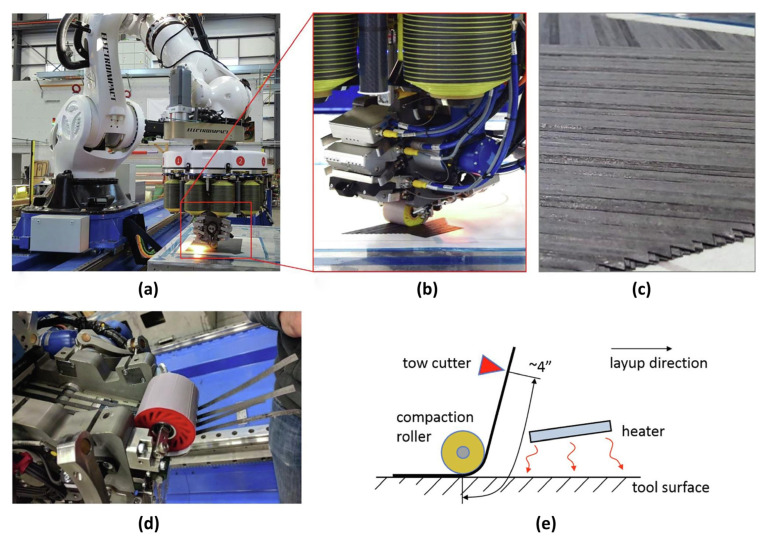
AFP manufacturing and parameters. (**a**) Robotic arm with modular head; (**b**) roller head during placement; (**c**) close-up of manufacturing; (**d**) slit-tapes being fed into roller head; (**e**) schematic of the layup process [47].

AFP utilizes robotic systems to precisely deposit prepreg composite tows onto a mold surface [45,46]. Although a mature conventional process, AFP’s inherently additive nature and high production efficiency make it irreplaceable for manufacturing large-scale aerospace primary structures (e.g., fuselage barrels, wing skins), as illustrated in Figure 9. It enables precise control of fiber placement paths, achieving highly tailored structural performance. Key challenges include substantial capital investment, complex programming requirements, and potential defects (e.g., wrinkling or overlapping) when conforming to highly contoured surfaces. Integration with in situ curing technologies to achieve out-of-autoclave (OOA) manufacturing constitutes an active research focus.

#### 3.2.8. Comparison of Techniques in Terms of Material Compatibility, Resolution, and Scalability

As Table 1 and Table 2 clearly delineate, the diverse landscape of CAM technologies offers aerospace engineers significant flexibility but also necessitates careful selection based on specific application requirements, as no single “optimal” solution exists universally. Technology readiness levels (TRLs) reveal stark disparities: established processes like FDM, SLM, and AFP are widely deployed industrially (TRL 7–9), while emerging methods such as DIW remain predominantly in the research domain (TRL 5–6). Economically, FDM excels for large secondary structures or interiors due to lower equipment costs and compatibility with certified thermoplastics, whereas capital-intensive systems like AFP or EBM incur significantly higher initial investments. Performance trade-offs are inherent and critical: high dimensional accuracy achievable with SLM or DIW often comes at the cost of reduced production efficiency and scalability; conversely, highly scalable processes like AFP or LMD exhibit constraints in dimensional precision and geometric complexity. Consequently, selecting the most suitable CAM technology demands a holistic evaluation integrating TRL maturity, economic viability (considering both capital and operational costs), and the specific performance priorities (e.g., precision vs. speed vs. scalability) dictated by the end-part function, geometry, volume, and cost targets.

## 4. Design Innovation and Performance Enhancement Through CAM in Aerospace

Building on the material systems and manufacturing processes for aerospace CAM introduced in the previous chapter, this chapter argues that advanced materials and methods alone are not enough to realize CAM’s full potential. True innovation requires a fundamental shift in design thinking. Section 4.1 shows how design for additive manufacturing (DfAM) exploits the unique properties of these materials and processes to break traditional manufacturing limits and enable new design approaches. Section 4.2 then examines how precise process control, informed by these design strategies, leads to optimized performance and successful fabrication of composite components.

### 4.1. DfAM Enabling Aerospace Innovation

Driven by continuous advancements in AM technologies, design methodology has evolved from the conventional “Design for Manufacturing” approach to a transformative paradigm of “Design with Manufacturing.” DfAM is a fundamental shift in design philosophy, integrating material properties, process constraints, and functional requirements from the project’s outset [56,57,58]. In the aerospace sector, this paradigm shift demonstrates particularly compelling momentum, as it directly addresses the industry’s dual imperatives for extreme performance and economic efficiency. The integration of mature CAE software (e.g., Nastran, Abaqus, Ansys) with composite-specific design platforms such as CATIA CPD and FiberSIM [59] delivers robust computational capabilities essential for implementing this highly integrated design workflow.

#### 4.1.1. Topology Optimization (TO)

TO, as an advanced design theory, provides a powerful impetus for innovative aerospace structural design [60]. Departing from reliance on engineers’ prior experience, it employs algorithms to identify optimal material distribution pathways within defined design spaces and loading conditions, thereby generating highly efficient, bio-inspired morphological structures that transcend conventional concepts of beams, ribs, and frames. Its significance lies in shifting design from a “form-driven” to a “performance-driven” paradigm, where material is placed only where needed to maximize mechanical performance.

**Figure 10:**
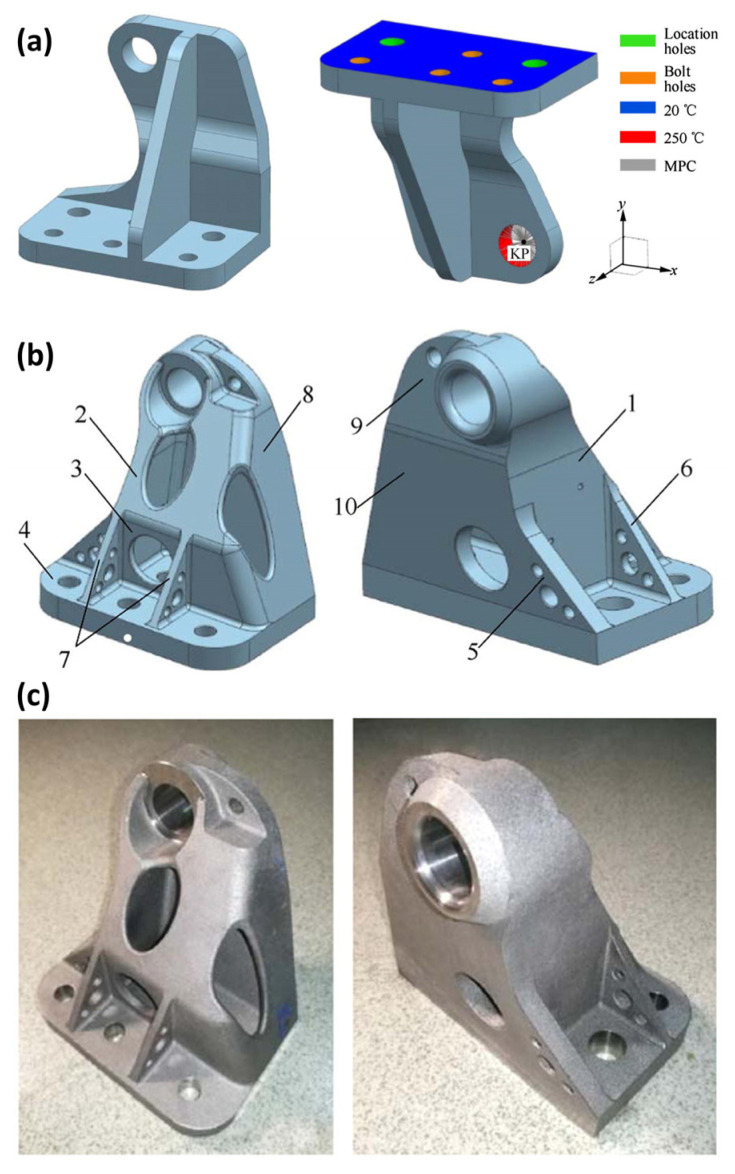
(**a**) Initial bracket design; (**b**) topology-optimized reconstructed bracket. Areas 1–10 represent critical structural regions identified based on engineering judgment and structural performance analysis. Their thicknesses serve as design variables and are adjusted during size optimization to achieve lightweight goals while ensuring structural safety; (**c**) as-manufactured component sample [61].

A prime exemplar is Airbus’s topology-optimized redesign of the A320 cabin bulkhead bracket. This approach achieved 45% weight reduction while maintaining or even enhancing component stiffness and strength [61]. For single-aisle aircraft, this magnitude of mass savings translates to tonne-scale fuel reductions over the operational lifecycle with commensurate CO_2_ emission cuts—directly addressing aviation’s dual imperatives of economic viability and environmental sustainability. This compellingly demonstrates TO’s role not merely as a mass-reduction tool, but as a strategic enabler for whole-lifecycle economic optimization of aircraft. Figure 10 illustrates the transformative progression of an aerospace bracket from initial design through TO to final manufacturing realization.

#### 4.1.2. Integrated Structure–Process Design

Integrated structure–process design shifts additive manufacturing (AM) constraints—such as minimum feature size, self-supporting angles, and thermal stress—from late-stage checks to key drivers in early design stages [62]. This means controlling slender features and avoiding enclosed cavities to minimize or eliminate support structures. The innovation lies in enabling cost-effective fabrication of topology-optimized “ideal shapes,” and even using process characteristics to introduce new functions.

For example, in spacecraft propulsion systems, printing fuel/coolant channels, valve interfaces, sensor mounts, and load-bearing structures directly onto thrust chambers or manifolds reduces leak risks, cuts weight by 20–40%, and shortens assembly time [63]. Beyond weight and cost savings, this high integration greatly improves system reliability by eliminating welds and mechanical joints.

**Figure 11:**
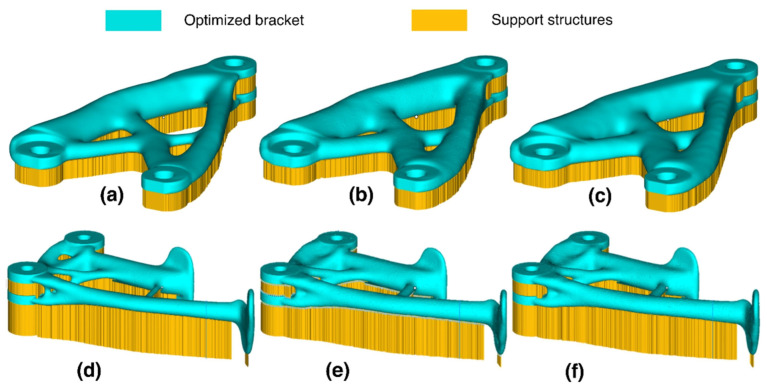
Support structure requirement for lightweight design of bracket A using (**a**) SIMP solution, (**b**) SIMP solution with ULS infill, and (**c**) SIMP solution with FGLS infill. Support structure requirement for lightweight design of bracket B using (**d**) SIMP solution, (**e**) SIMP solution with ULS infill, and (**f**) SIMP solution with FGLS infill [64].

Figure 11 compares support requirements across three design strategies. SIMP produces complex overhangs and needs extensive support. Uniform lattice structures (ULSs) reduce support needs by partially supporting themselves. Functionally graded lattice structures (FGLSs), with gradient-based designs that avoid overhangs, require the least support. Table 3 provides quantitative comparison data.

Table 3 reveals a critical trade-off: while the FGLS strategy yields the greatest theoretical weight savings (52.5%), it incurs a severe penalty in manufacturability, evidenced by a dramatic increase in required support area and post-processing time. Consequently, the balanced nature of the ULS solution (delivering a 40.01% weight reduction while maintaining controllable support requirements) further substantiates that embedding process constraints upfront enables effective co-optimization of cost, performance, and manufacturability.

#### 4.1.3. Self-Supporting Structures

In AM, overhanging components typically require additional support structures, which not only reduce printing efficiency but also introduce post-processing complications upon removal. Self-supporting design has thus emerged as a critical research focus, enabling AM to eliminate dependency on supports. A universal constraint in industrial processes (e.g., SLM or EBM) mandates that downward-facing surfaces (overhangs) maintain an inclination angle exceeding a critical threshold relative to the build plate. Components satisfying this criterion across all geometries are deemed self-supportable in the given build orientation. This fundamental AM constraint has been extensively studied across technologies [65,66,67], with the critical angle typically approximating 45 degrees. Components containing features below this threshold in their intended build direction cannot achieve self-support and thus cannot be printed directly. Therefore, Method 1 involves modifying the component’s shape. The drawback of this approach is that altering the optimized geometry may lead to performance degradation or even render the design infeasible. Method 2 incorporates temporary support structures during manufacturing, which are subsequently removed through conventional post-processing steps. The disadvantage of this method is that printing and subsequent removal of supports increase material consumption, printing time, and post-processing costs.

**Figure 12:**
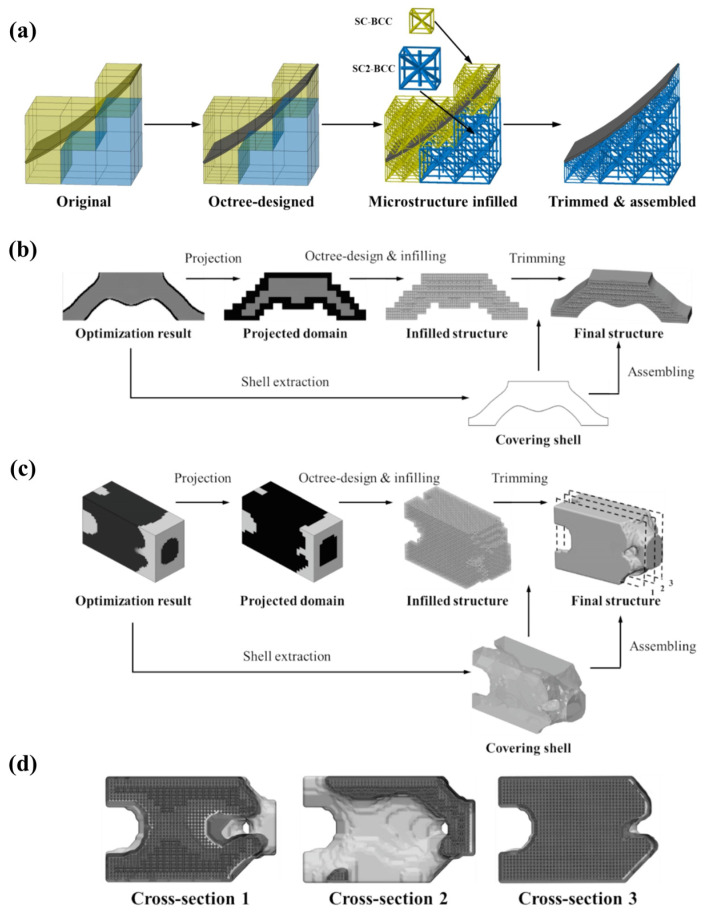
(**a**) Self-supporting design for shells in lattice-filled structures; (**b**–**d**) dehomogenization reconstruction of homogenized optimization results: (**b**) three-point bending beam case; (**c**) cantilever beam case; (**d**) gradient feature distribution across three sections of the cantilever beam case [70].

For aerospace applications, eliminating support structures translates to reduced material consumption and significantly shortened post-processing time; self-supporting structures enable implementation of extremely thin walls, fine mesh/lattice structures, and large-span cantilever structures, which in conventional designs are either unmanufacturable or require excessive reinforcement leading to weight gain. The self-supporting lattice infill design method demonstrated in Figure 12a utilizes boundary-adaptive octree subdivision technology to enable the internal lattice core to autonomously support the top shell during manufacturing. This design directly eliminates the need for additional support structures in traditional AM, significantly reducing material consumption and substantially shortening post-processing time. More importantly, the self-supporting capability grants greater design freedom to structural configurations; as illustrated, the optimized lattice structure can generate sub-millimeter wall thicknesses, refined non-uniform lattices, and large-span cantilever geometries (cantilever beams).

Langelaar [68] proposed a density-driven compatibility formulation that overcomes unprintable designs and related inefficiencies. By strictly enforcing overhang angle constraints applicable to the target AM process at every step of the optimization procedure, self-supporting, fully printable designs are achieved. Scholars such as Mezzadri et al. [69] successfully generated complex structures requiring no or minimal supports by introducing overhang angle constraints into TO algorithms. For aerospace applications, the value of this design philosophy is immense. It not only directly reduces material and labor costs but, more significantly, enables the manufacturing of ultralight structures featuring fine lattices, thin walls, and large-span cantilever geometries—structures that in conventional designs are either unmanufacturable or require excessive reinforcement leading to weight gain.

#### 4.1.4. Computer-Aided Design

Integrated with computer-aided design (CAD) software, AM achieves rapid transformation from digital models to physical products, a process that significantly advances design innovation and manufacturing flexibility. CAD software enables users to create precise three-dimensional models that can be directly utilized in the AM process, thereby reducing the time and costs required by traditional manufacturing techniques. This integrated workflow supports high-level customization and personalized production, allowing even complex geometric structures to be feasibly printed [71].

Zhang et al. [71] presented a geometry-based computational preprocessing algorithm for powder bed fusion AM processes within a CAD framework. Their developed algorithms and tools were successfully demonstrated on two sample components. Chen et al. [72] demonstrated the development of safety features in CAD files using curvature and internal surfaces.

### 4.2. From Design to Reality: Optimization and Realization of Physical Properties

**Figure 13:**
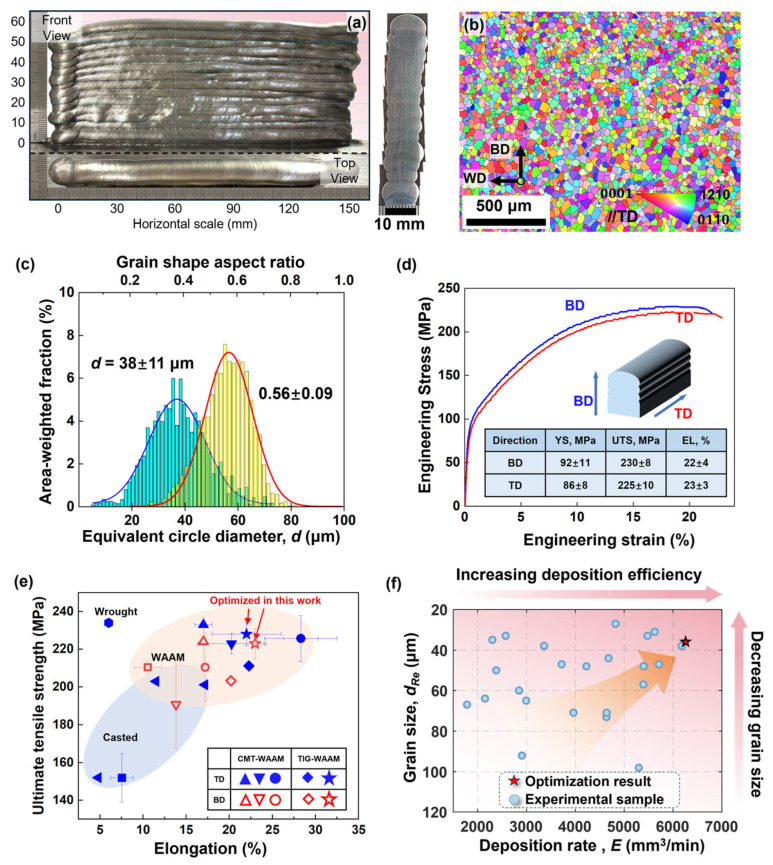
Macroscopic dimensions and microstructure of deposits fabricated with optimized process parameters: (**a**) macroscopic morphology; (**b**) matrix grain orientation distribution (IPF map); (**c**) grain size distribution and grain aspect ratio distribution; (**d**) room temperature tensile properties; (**e**) tensile property comparison with existing studies; (**f**) deposition efficiency and grain size comparison between optimized results and experimental samples [80].

If DfAM addresses the strategic question of “what shape to manufacture,” this section focuses on the tactical challenge of “how to achieve optimal performance.” An exceptional design blueprint must be transformed into a high-performance physical entity through precise control of the manufacturing process. Gu et al. [73] proposed that laser AM holds potential to revolutionize component design by enabling cross-scale regulation, reducing manufacturing steps, and expanding viable structures for end-use applications. In metal AM, its unique heating/cooling cycles create fine microstructures distinct from conventional metal processing techniques, thereby conferring superior mechanical [74,75,76] and electrochemical [20,77,78] properties. For particle-reinforced titanium matrix composites, studying reinforcement dissolution/reactions, precipitate evolution, and in situ synthesis mechanisms enables interfacial bonding control between reinforcement and matrix, enhancing composite hardness, strength, and ductility. Furthermore, Yakout et al. [79] demonstrated that AM can optimize microstructures via precise parameter control to tailor mechanical properties. As shown in Figure 13 for wire arc additive manufacturing (WAAM) of AZ31 magnesium alloy, the optimized process yielded a refined, homogeneous, fully equiaxed grain structure with an average grain size of 38 μm (Figure 13b,c), resulting in components exhibiting an ideal combination of high strength and excellent plasticity (ultimate tensile strength ~230 MPa, elongation >22%). Moreover, mechanical properties showed high consistency along both the building direction (BD) and transverse direction (TD) (Figure 13d), demonstrating exceptional isotropy. Figure 13e further highlights the competitiveness of the optimized results, with their strength–ductility balance surpassing that of numerous literature-reported cast, forged, and WAAM-processed AZ31 alloys. Figure 13f then visually illustrates the superior position of the optimized point (high deposition efficiency + fine grains) within the parameter space, significantly outperforming other experimental samples. Crucially, AM transcends a simple “printing” process to constitute a controllable “metallurgical” or “solidification” process, enabling in-process performance optimization.

#### 4.2.1. Precise Control of Material Microstructures

The unique thermal process of AM (rapid heating and cooling) provides a powerful lever for precise control of material microstructures. By meticulously adjusting process parameters such as laser/electron beam power, scanning speed, and scanning strategy, one can directly influence melt pool dimensions, temperature gradients, and cooling rates, thereby governing the final component’s grain size, morphology, orientation, and phase distribution [79]. For instance, during MMC fabrication, optimized parameters can suppress brittle phase formation and promote robust metallurgical bonding between reinforcement and matrix [27]. For high-performance polymer matrix composites like PEEK, precise temperature control optimizes crystallinity, significantly enhancing mechanical properties and dimensional stability [22]. This capability to “sculpt” material performance at the microscale constitutes the fundamental advantage of CAM over traditional manufacturing for performance customization.

#### 4.2.2. Bio-Inspired Structures

**Figure 14:**
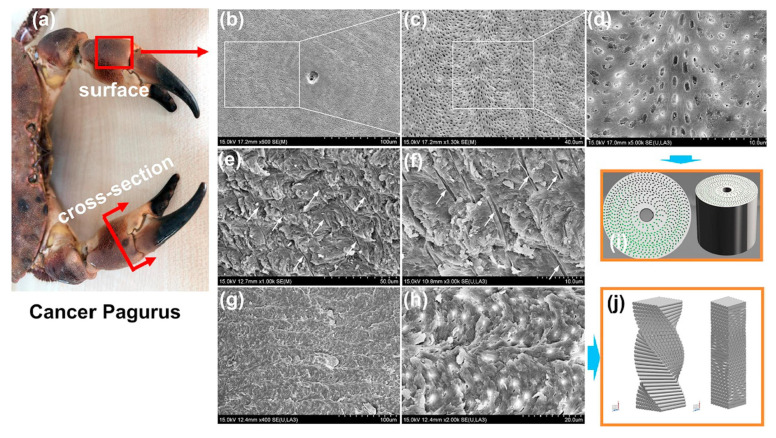
(**a**) Macroscopic morphology of Cancer pagurus; (**b**–**d**) surface morphological features of the edible crab under varying magnifications, showing helical distribution of abundant pores; (**e**,**f**) fracture structure characteristics of crab cross-sections at different magnifications; (**g**,**h**) microstructural features of polished cross-sections; (**i**) schematic of porous structure and (**j**) generalized 3D model of helical architecture [81].

Bio-inspired structures, drawing inspiration from the ingenious architectures of flora and fauna, typically exhibit exceptional mechanical properties. Simultaneously, such structures emulate the natural design principles that sustain biological functions, enabling multifunctional capabilities. The integration of geometric customization with AM offers novel opportunities for developing high-performance architected lattices. Geometric customization of honeycomb structures can be achieved by linearly varying cell wall thicknesses along the full-thickness direction while maintaining overall structural mass. Cancer Pagurus’s claw has been studied for its helically distributed pore channels and characteristic spiral structure (Figure 14). These features not only endow the crab claw with superior mechanical properties but also inspire AM designs. Results demonstrate [81] that three distinct pore distribution patterns based on the crab claw design significantly enhance toughness in SLM-fabricated Ni-Ti components while exhibiting comparable energy absorption capacities.

#### 4.2.3. Path Optimization

Path planning significantly influences forming efficiency, precision, and the microstructural evolution of materials. In practical applications, AM technologies enable the production of high-performance functionally graded materials, multi-material integrated components, and near-net-shape parts through laser-directed energy deposition. This demonstrates the critical role of path planning in achieving complex structural and functional integration. Jin et al. [82] proposed a toolpath generation method for material extrusion-based AM that balances processing efficiency and manufacturing accuracy, specifically addressing the generation of direction-parallel toolpaths for internal filling of simple connected regions; the results demonstrate its effectiveness and distinct advantages. Jiang et al. [83] introduced a novel support generation strategy incorporating internal/external support considerations within AM process planning to reduce material consumption, production time, and energy usage; the comparative results confirm substantial reductions in material waste, production time, and energy consumption versus conventional approaches.

#### 4.2.4. Improvement of Mechanical Properties

AM has demonstrated significant potential for enhancing the mechanical properties of aerospace composites by enabling precise control over the distribution and orientation of reinforcing phases (e.g., continuous carbon fibers, ceramic particles, or whiskers), thereby achieving performance optimization unattainable through conventional processes. Research indicates that AM-fabricated continuous fiber-reinforced polymer matrix composites (such as nylon/carbon fiber systems) can attain tensile strengths exceeding 800 MPa and elastic moduli above 60 GPa—representing a 300–500% improvement over traditional injection-molded parts—while maintaining densities below 1.3 g/cm^3^, significantly optimizing specific strength [84]. For CMCs (e.g., SiC fiber-reinforced SiC), AM processing increases fracture toughness by 30–50%, effectively suppressing crack propagation [85] and meeting the stringent damage tolerance requirements of combustion chamber liners. In MMCs (e.g., aluminum-based nano-ceramic reinforced systems), AM achieves uniform dispersion of nanoparticles, yielding strengths surpassing 500 MPa while retaining elongations above 8% [86]. Furthermore, AM’s unique capability for gradient structural design allows composites to achieve improvements of 16.9% in elastic modulus, 39.8% in yield strength, and 70.1% in compressive strength when comparing 0° and 90° gradient orientations [87]. Copper–graphene composite films prepared via electrochemical deposition exhibit thermal conductivity of 460 W/mK at 300 K for thicknesses >200 μm, marking a 21% enhancement over pure copper (380 W/mK) [88]. These data confirm that AM technology, through precise microstructural control, delivers synergistic leaps in strength, modulus, toughness, and functionality for aerospace composite materials.

## 5. Aerospace Applications and Recent Innovations in CAM

### 5.1. Current Aerospace Applications of CAM

Current applications reveal a clear adoption trajectory for CAM in aerospace: progressing from interior to exterior components, from non-load-bearing to primary structures, and from simple parts to integrated, multifunctional systems. This path reflects a prudent, risk-based strategy, prioritizing applications with less stringent certification requirements before expanding into mission-critical domains.

#### 5.1.1. Structural Components

**Figure 15:**
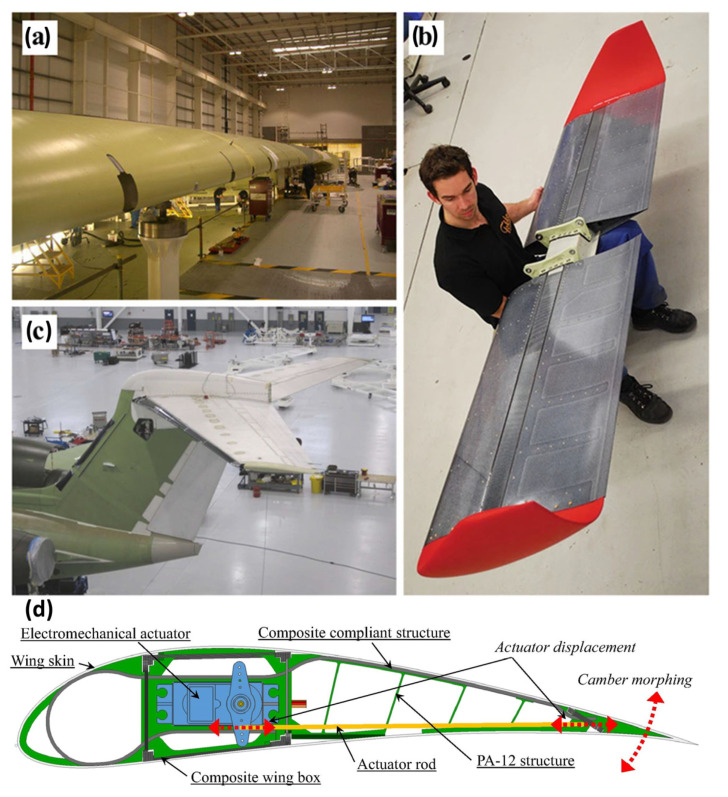
(**a**) Airbus A380 fixed-wing J-nose leading edge; (**b**) Agusta Westland AW169 helicopter empennage assembly; (**c**) elevator and rudder installed on a Gulfstream prototype aircraft; (**d**) airfoil camber morphing concept based on compliant composite structures and electromechanical actuators [89,90].

Fiber-reinforced polymer composites are preferred for structural components like fuselage skins and spars due to their high specific strength and design flexibility. Processes such as automated tape laying (ATL) enable the integrated manufacturing of complex curved structures, achieving 20–30% weight reduction over metallic counterparts and improving fuel efficiency. For example, the fuselage sections of the Boeing 787 and Airbus A350 utilize carbon fiber composites, balancing structural efficiency with fatigue resistance; the Airbus A350 and Boeing 777X incorporate continuous fiber-reinforced nylon matrix additively manufactured composite parts to achieve lightweighting and high strength [89,90]. Figure 15 illustrates structural components applied to different aircraft types (Figure 15a–c) and the airfoil camber morphing concept (Figure 15d).

#### 5.1.2. Interior Components

**Figure 16:**
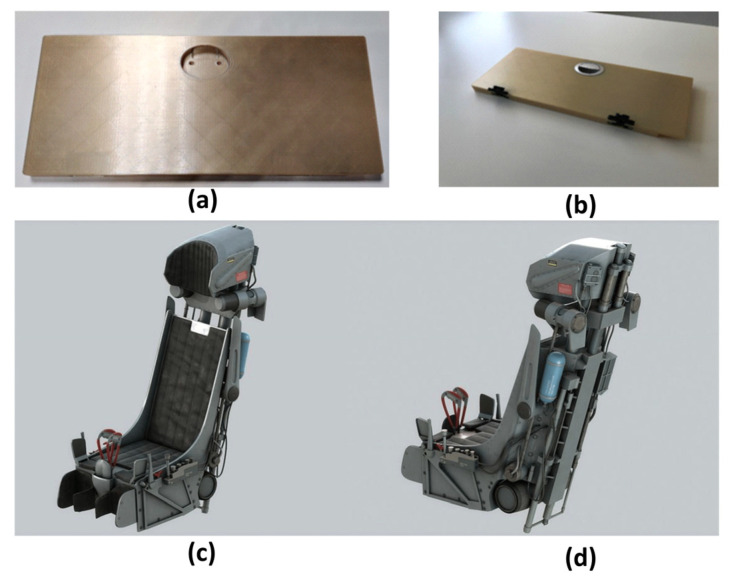
Folding tray table 3D-printed with Ultem 9085 material (**a**) and assembled with mounting points (**b**). Front view (**c**) and rear view (**d**) of the 4th generation K-39 ejection seat [91,92].

High-performance polymers such as Ultem 9085 and PEEK are processed via FDM for interior components like armrests and panels. These materials meet aviation safety standards for high-temperature resistance and low smoke toxicity. AM also facilitates complex curved designs that improve passenger comfort and space utilization. Boeing employs titanium alloy AM to produce aircraft seat brackets, achieving significant weight reduction compared to conventional processes while improving structural strength [91,92]. Figure 16 displays a tray table fabricated with Ultem 9085 material (Figure 16a,b) and a military seat incorporating metal AM (Figure 16c,d).

#### 5.1.3. Engine Components

**Figure 17:**
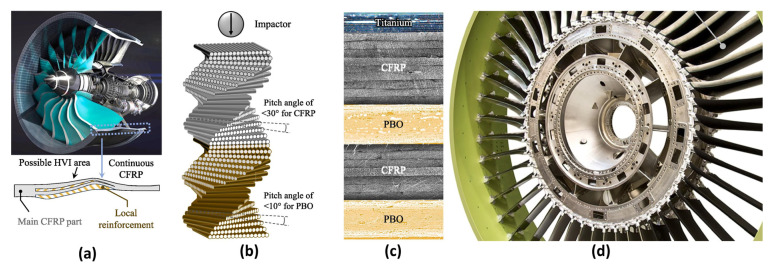
(**a**) A cut of an aircraft engine showing its containment casing cross-section and the corresponding schematic at the bottom, (**b**) bio-inspired helicoidal layup and (**c**) hybrid interleaved designs. (**d**) Composite fan case demonstrator developed by GKN Aerospace Sweden [95,96].

MMCs are used for engine nacelles and turbine ducts, enabling operation above 800 °C due to their high-temperature resistance and low CTE. Nozzles fabricated from SiC/Ti composites, for example, achieve a theoretical 40% weight reduction and enhanced creep resistance compared to nickel-based superalloys [93]. The low wear rate demonstrated by aluminum matrix composites in piston ring–cylinder liner friction pairs also supports their potential application in aero-engine sliding components [94]. Figure 17 presents a cross-sectional schematic of an aircraft engine, illustrating a bio-inspired helicoidal layup design and hybrid interleaved design, along with a turbine engine blade fabricated from carbon fiber-reinforced polymer (CFRP) material.

#### 5.1.4. UAVs and Satellites

AM technology achieves integrated lightweight design and high strength for UAV components through TO and lattice structure design. Regarding flight endurance, CFRP composites formed via FDM or SLS technologies further reduce structural mass, extending UAV operational duration. For wing structures, selective material deposition enables UAV wings to concurrently provide structural support, electromagnetic shielding, and thermal management functions.

In satellite applications, Made In Space’s Archinaut system performs in-space fabrication of carbon fiber-reinforced truss structures with radiation-resistant properties superior to those of traditional aluminum alloys. Composite pressure vessels (e.g., fuel tanks for hybrid rocket engines) achieve integration of lightweight design and high sealing performance through additive filament winding technology [97].

### 5.2. Case Studies of Successful Implementations by Companies

**Figure 18:**
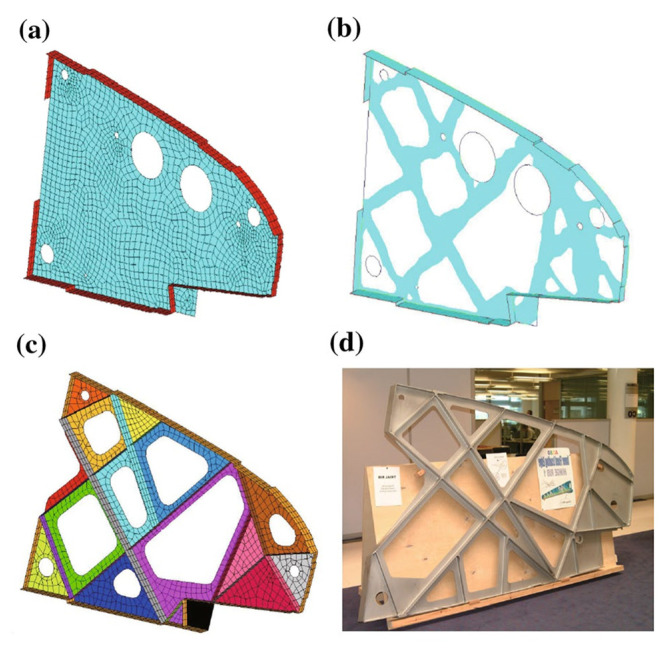
(**a**) Design space and non-design space regions; (**b**) TO recommended design; (**c**) initial design for dimensional and shape optimization; (**d**) prototype machined from high-strength aluminum alloy [99].

NASA Johnson Space Center and Oak Ridge National Laboratory have investigated the potential of AM for heat shields using thermoplastic materials via filament-based 3D printing, marking the first demonstration of AM fabrication and assembly for thermal protection in space systems. Airbus leveraged TO and AM to redesign the A320 cabin hinge bracket [98], representing a practical application case of metal AM technology in commercial aircraft interior components. Figure 18 illustrates the topology, dimensional, and shape optimization design process for the A380 cabin hinge bracket.

When confronting manufacturing requirements for large dimensions, high precision, and superior performance, selecting appropriate AM technologies (e.g., full additive manufacturing [100]) and process control methods (such as the planar deposition method [101]) becomes critical. Integrating TO with AM technologies transcends the limitations of conventional manufacturing processes, enabling efficient production of complex structures [102].

### 5.3. Breakthrough Innovations in Aerospace CAM

#### 5.3.1. Continuous Fiber-Reinforced Composite AM for High-Performance Aerospace Parts

**Figure 19:**
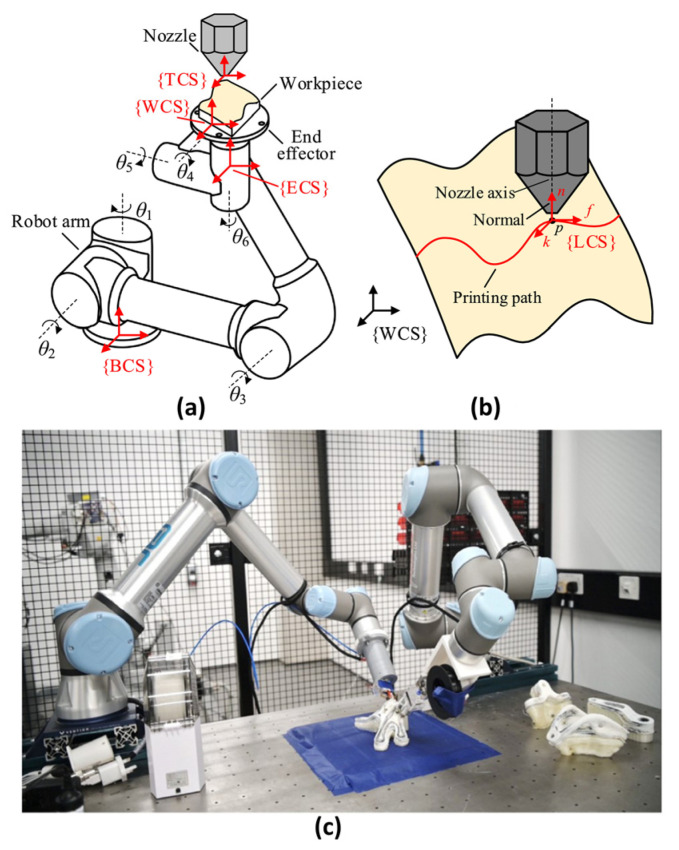
(**a**) Schematic of robotic continuous long-fiber deposition modeling (CLFDM) system; (**b**) deposition path within the work coordinate system (WCS) reference frame; (**c**) dual-robotic-arm setup manufacturing continuous fiber-reinforced thermoplastic composites (CFRTPCs) with spatially variable fiber orientations in 3D space [104,105].

This technology aims to achieve mechanical properties in AM components that approach or even surpass those of traditional autoclave processes through precise continuous fiber placement during printing, representing one of the most critical advancements in CAM (with current TRL ≈ 6–7). For fiber orientation control, variable deposition direction (VDD) technology optimizes fiber paths via mathematical models to dynamically adjust deposition trajectories. Experimental results demonstrate a 69.4-fold enhancement in tensile performance for VDD-fabricated hollow cylindrical structures compared to unidirectional counterparts [103]. Robotic multi-axis collaborative printing, integrated with 6-DoF manipulators, enables continuous fiber-aligned deposition on curved surfaces, overcoming limitations of planar layer-by-layer fabrication, as shown in Figure 19. However, two fundamental bottlenecks hinder its large-scale adoption: firstly, low deposition rates lead to prohibitively high costs that cannot compete with mature AFP technology; secondly, the absence of robust in-process quality monitoring and non-destructive inspection methods compromises manufacturing consistency for primary load-bearing structures and ultimate flight safety certification.

In the field of continuous fiber FDM technology, the in-melt feeding process achieves a fiber volume fraction of 50% through parameter optimization, offering a novel high-precision, high-strength lightweight manufacturing pathway for load-bearing thermoplastic composite structures in aerospace applications [106]. For aeronautical primary structures, robotic multi-axis AFP alternatives must achieve deposition rates >1 kg/h to compete with autoclave processes, while meeting FAA certification requirements for defect tolerance [107]. Concurrently, the “On-Component Embedding Method,” which integrates fiber surface modification (PVA treatment) with real-time cooling processes, significantly enhances the interfacial bonding strength of carbon fiber/PLA composites, providing a customizable manufacturing solution with high flexural performance for aerospace hot-end components [108].

For interfacial bonding enhancement, in situ curing technology enables synchronous thermosetting of resin polymerization within dual-material systems during printing, reducing porosity while improving fiber matrix interfacial strength. Meanwhile, nano-reinforced interfacial layers incorporating silica nanoparticles or carbon nanotubes as transition phases augment bonding efficacy through dual mechanisms of mechanical interlocking and chemical bonding.

For thermal stress management, in-process infrared thermography enables real-time monitoring of temperature distribution during fabrication. Integrated with machine learning algorithms, this approach predicts thermal stress concentration zones, effectively reducing interlaminar residual stresses and diminishing warpage deformation.

#### 5.3.2. Development of New Materials

**Figure 20:**
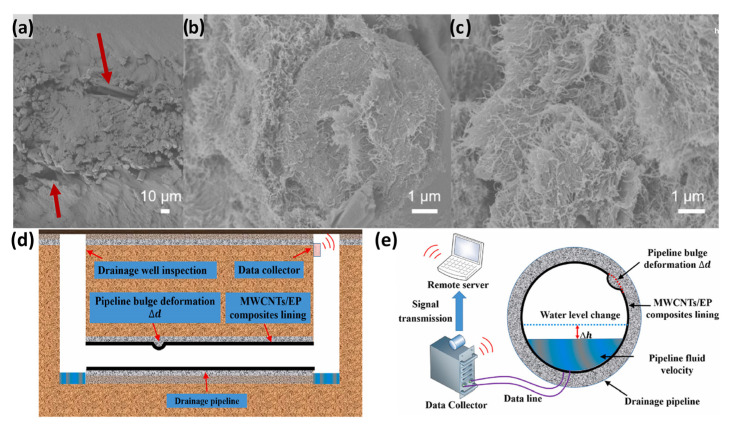
(**a**) Fracture surface of nylon with integrated conductive layer (CL); (**b**,**c**) micrographs of carbon fibers and carbon nanotubes in epoxy adhesive matrix, respectively; (**d**) system-level view of MWCNT/EP composite deployment; (**e**) detailed perspective of MWCNT/EP composite monitoring pipeline deformation [111,112].

The development of AM-specialized composite materials with tailored functionalities—such as high-temperature resistance, self-healing capabilities, and sensing properties—serves as the primary driver for expanding CAM application boundaries (most research in this domain resides at TRL 4–6). High-temperature resins (e.g., polyimides, bismaleimides), with Tg exceeding 300 °C, constitute core materials for extreme environments in aerospace and energy systems [109]. Kathy et al. [109] successfully fabricated short carbon fiber-reinforced polyimide components via SLS, achieving a post-cured Tg of 370 °C suitable for high-temperature aviation applications. Meanwhile, multi-walled carbon nanotube (MWCNT)/epoxy composites (1 wt.%) processed by ultrasonic dispersion demonstrated high strain sensitivity coefficients as piezoelectric sensors for structural health monitoring [110]. Key challenges include narrow processing windows for novel material systems and insufficient long-term performance data (e.g., fatigue resistance, hydrothermal aging resistance), resulting in protracted and costly airworthiness certification cycles. Figure 20 presents tensile test results of piezoresistive sensors embedded in nylon matrices for structural health monitoring (Figure 20a–c) alongside schematic representations of MWCNT/EP composites for pipeline deformation monitoring (Figure 20d,e).

#### 5.3.3. Large-Scale Composite AM for Manufacturing Large Aerospace Structures

This technology (TRL 7–8) fabricates meter-scale monolithic structures to reduce assembly, minimize mass, and enhance integrity. Large-format CAM is now transitioning to practical applications for primary structures, and its capability to produce large, high-performance integrated components has been validated. As a core enabling process, AFP demonstrated significant value in Boeing’s practice: the company successfully manufactured a 5.5 m diameter composite fuel tank using AFP systems. This achievement not only confirms AFP’s viability for large pressure vessels but also establishes its reliability in meeting stringent aerospace performance requirements [113]. Concurrently, major breakthroughs have been achieved in thermoplastic composite-based large-format AM for beam structures like airframes and wings. COMAC leveraged Farsoon’s Flight™ technology to fabricate an 8.2 m long continuous carbon fiber-reinforced PAEK composite wing spar for its C929 wide-body aircraft prototype. These accomplishments mark milestone advancements in large-scale AM for producing extreme-dimension, highly complex integrated structures. Critical bottlenecks include maintaining dimensional accuracy while mitigating residual stresses and distortion induced by heat accumulation during ultra-large builds, alongside unresolved engineering challenges regarding equipment stability and long-duration continuous operation reliability.

#### 5.3.4. Integration of Sensors and Smart Materials into Composite AM Components

**Figure 21:**
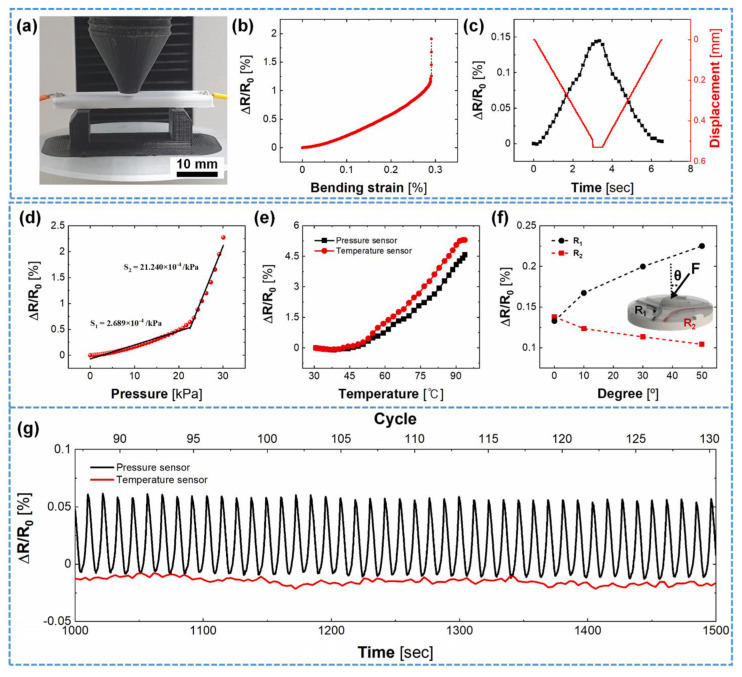
(**a**) Bending test of 3D-printed beam structures; (**b**) relative resistance change versus bending strain curve for single-beam configuration; (**c**) loading–unloading cycles of single-beam structure; (**d**) relative resistance change versus applied pressure curve for pressure sensor, the relative resistance change can be divided into two linear regions, with the red curve representing the measured values and the black line showing the result of the linear fit; (**e**) relative resistance change versus temperature profiles for pressure-sensing and temperature-sensing units; (**f**) relative resistance variation of pressure-sensing units under 9.8 N force application at different angles; (**g**) relative resistance response of sensor under 5 N applied force [118].

Integrating sensing, actuation, and other functionalities directly into composite structures represents the ultimate objective for structural–functional integration and intelligent flight vehicles (current TRL predominantly at 4–5 laboratory validation stages). FDM enables direct printing of sensors using polymer matrices (e.g., ABS, TPU) filled with carbon nanotubes (CNTs), graphene, or carbon black. For instance, 3D-printed conductive carbon black sensors detect strain and porosity variations with gauge factors (GFs) of 15–20, surpassing silicon-based sensors [114]. LMD and ultrasonic additive manufacturing (UAM) can embed fiber Bragg grating sensors within metallic matrices for distributed temperature/strain monitoring, offering electromagnetic interference immunity and high-temperature endurance [115,116]. Figure 21 exhibits multifunctional performance metrics of sensing materials—fabricated from 3 wt% MWCNT/PLA composite filaments—integrated within beam structures, demonstrating the developmental trajectory of multifunctional composites.

In smart material system innovations, the National Composites Centre (NCC) UK has developed a microcapsule-based self-healing system. By co-printing dicyclopentadiene (DCPD) microcapsules (50–200 μm diameter) with carbon fiber/PA6 composites, it achieves 92% crack-healing efficiency [117]. Additionally, composites embedded with magnetic or photothermal nanoparticles demonstrate field-driven shape-morphing capabilities under external magnetic fields or light exposure, enabling targeted drug delivery and soft robotics actuation.

The primary barriers to industrialization lie in the following: interfacial compatibility between embedded sensors and matrix materials coupled with long-term service reliability challenges and the absence of established standards for signal interpretation, data processing, and health diagnostics of embedded sensing systems—preventing their seamless integration into aircraft condition monitoring and predictive maintenance systems.

#### 5.3.5. Technology Integration and Future Impact

In manufacturing, embedded sensors can provide real-time data foundations for digital twins; however, aviation airworthiness certification scenarios require additional breakthroughs in key challenges such as high-temperature attenuation (>300 °C) and electromagnetic interference suppression [119]. Recycling of thermoset matrices (e.g., pyrolysis achieving >90% strength retention) combined with large-scale manufacturing can achieve significant carbon reduction (GWP −23.6 kg CO_2_ eq/kg). Nevertheless, processes like fluidized bed methods must improve current 50–82% strength retention rates to >90% to maximize environmental benefits [120]. Through sustainability-driven design approaches (e.g., comprehensive SI metric optimization), aerospace composite components can integrate high proportions of recycled materials (≥30%) without significant performance sacrifice, providing a technical pathway for the EU Clean Aviation initiative’s recycling targets [121].

## 6. Bottlenecks, Trade-Offs, and Sustainability: Real-World Challenges for Aerospace CAM

While the preceding chapters demonstrated CAM’s potential, its transition to widespread industrial feasibility is impeded by several interlocking challenges. This chapter critically examines these core bottlenecks and analyzes key sustainability imperatives, providing an empirical foundation for the subsequent discussion of future research.

### 6.1. Overarching Challenges in Aerospace CAM

**Figure 22:**
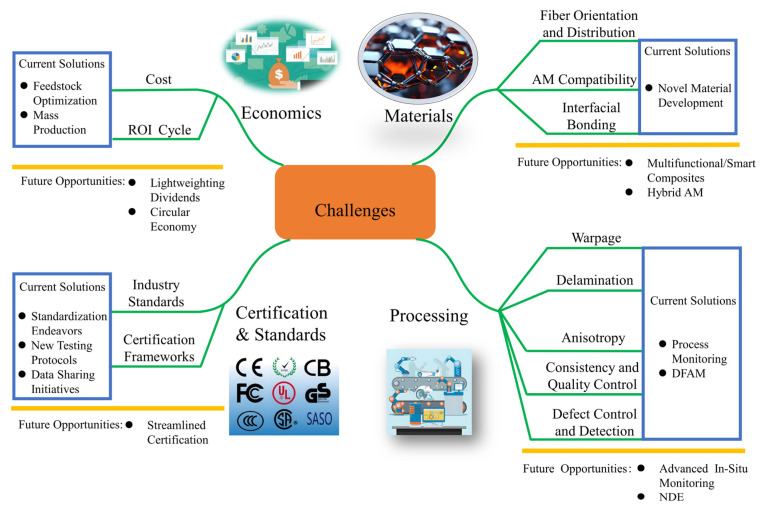
Key challenges in AM.

The challenges in aerospace CAM are not isolated; they form an interdependent network where material limitations, process uncertainties, and certification complexities are mutually reinforcing (Figure 22).

#### 6.1.1. Material Challenges

The current range of AM-compatible composite systems remains limited, fundamentally constraining design freedom and performance ceilings [13]. Within CAM, the restricted availability of AM-compatible materials constitutes a critical bottleneck impeding technological advancement. Presently, the market suffers from severe scarcity of composite materials suitable for mainstream AM processes (e.g., fused deposition modeling, selective laser sintering), primarily due to complexities in material–process compatibility and performance co-optimization. AM technologies impose unique requirements on material rheological properties and thermal response behaviors. For CMCs, traditional MAX-phase materials (e.g., Ti3AlC2/SiC) demand precise control of powder/polymer ratios (typically 85:15 to 70:30) and sintering temperatures (1300 °C optimal) during melt-based processing to achieve controllable porosity between 5 and 15% [122]. Meanwhile, natural fiber-reinforced polymers exhibit >30% reduction in tensile strength compared to compression molding counterparts when processed via FDM, primarily due to fiber fracture and interfacial debonding.

Introducing fiber reinforcements into AM processes typically aims to enhance mechanical properties, yet simultaneously introduces critical challenges in fiber orientation and distribution control. Unlike conventional manufacturing, AM’s layer-wise deposition induces anisotropic microstructures due to shear-induced alignment during extrusion and post-deposition cooling effects. This anisotropy directly governs key performance metrics—including tensile strength, thermal conductivity, and fatigue resistance—rendering precise fiber orientation control essential for performance optimization. Complex geometries (e.g., sharp corners, overhangs) disrupt flow-field uniformity, triggering localized fiber agglomeration or orientation deviations.

Aerospace components withstand extreme environments, including high stress, elevated temperatures, cyclic loading, and corrosion, imposing exceptionally stringent requirements on internal material integrity and interfacial quality. MMCs widely used in such applications face amplified difficulties in achieving desirable melt pool fluidity and metallurgical bonding due to inherent characteristics: high melting points, specialized thermophysical properties, and oxygen sensitivity. For diverse material combinations, physicochemical compatibility presents significant challenges. Dissimilar metal pairs—such as titanium alloys with stainless steel or nickel-based alloys with copper alloys—encounter poor mutual solubility and brittle phase formation. The spreading capability of molten metal on solid substrates directly influences fusion width and bonding effectiveness [123]. These material-level uncertainties fundamentally cause performance variations in final products compared to conventionally manufactured counterparts.

#### 6.1.2. Process Challenges

Warpage primarily stems from thermal stress accumulation and non-uniform shrinkage during AM processes. In techniques such as FDM, rapid cooling following layer-by-layer material deposition induces interfacial stress concentration due to mismatched coefficients of thermal expansion between the matrix and reinforcement phases. When material cooling rates exceed critical thresholds, localized shrinkage differentials can surpass 30%, significantly exacerbating warpage. Additionally, overhanging features or large-span regions in structural designs are more prone to deformation due to insufficient support mechanisms.

Delamination originates from inadequate interlaminar bonding strength, primarily caused by subsequent deposition layers inducing partial remelting–recrystallization of cured layers under high temperatures, forming weak interfaces; misalignment of continuous fibers at interlayer transition zones creating stress concentration points that trigger interfacial debonding; and contamination from air bubbles or unmelted particles during printing.

Anisotropy manifests primarily as directional variations in mechanical, thermal, or electrical properties, originating directly from the layer-wise deposition nature of AM, fiber orientation patterns, and process parameter settings. In FDM, short carbon fibers tend to align with nozzle movement direction, resulting in significantly higher tensile strength parallel to the printing orientation compared to the perpendicular direction, thereby creating pronounced anisotropy [23]. Furthermore, inhomogeneous interlayer thermal histories during layer-by-layer deposition generate localized temperature gradients, inducing differential shrinkage within the resin matrix and residual stress accumulation, which further induces anisotropic behavior.

**Figure 23:**
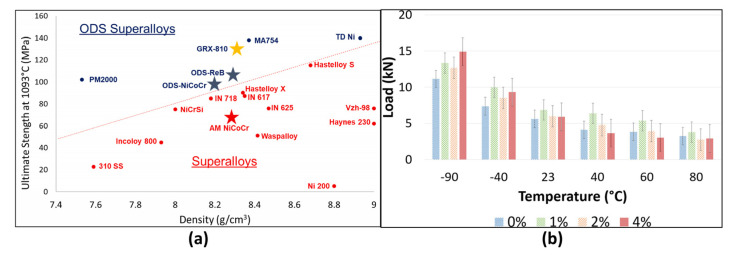
(**a**) Comparative analysis of ultimate tensile strength versus density for additively manufactured GRX-810 alloy against other oxide dispersion-strengthened (ODS) alloys and nickel-based superalloys at elevated temperature (1093 °C). (**b**) Relationship between elastic modulus and both temperature and nanoparticle content when compositing ceramic nanofillers (Aerosil 300) with polycarbonate (PC) polymer matrix [63,126].

Technically, material consistency and quality control during AM constitute critical challenges [124]. Ensuring reliability and performance of every component—particularly in extreme environments—remains imperative [63]. Altering material systems and manufacturing methodologies presents formidable challenges necessitating robust quality management systems and facility certifications (e.g., Nadcap, AS9100) [125]. AM technologies currently lag behind conventional processes in production speed and yield, constraining large-scale industrial implementation. NASA’s GRX-810 oxide dispersion-strengthened nickel-based alloy (Figure 23a) exhibits significant strength advantages at 1093 °C, substantially outperforming traditional alloys such as Inconel 718. However, this performance is critically dependent on precise AM process control: uniform dispersion of nano-yttria (Y_2_O_3_), stable laser melt pool dynamics, and subsequent hot isostatic pressing (HIP) treatment. Minor deviations induce fluctuations in grain boundary strengthening efficacy, directly compromising high-temperature creep life. Similarly, Figure 23b reveals inconsistent elastic properties of Aerosil/polycarbonate composites under thermal cycling conditions.

During processing, the narrow and highly sensitive process parameter window poses significant challenges: insufficient or uneven energy input readily causes lack-of-fusion porosity or poor inter-track bonding, while excessive energy density triggers keyhole instability, leading to deep penetration voids or spherical gas pores from entrapped shielding/powder-adsorbed gases [127]. The rapid melt–solidification process generates severe thermal gradients and contraction stresses—particularly at locations with significant CTE mismatches or geometric constraints (e.g., thin walls, sharp corners)—that frequently exceed local material strength limits, initiating hot cracks. For sensitive materials like certain superalloys, repeated thermal cycling may further promote deleterious phase transformations or impurity segregation in heat-affected zones, inducing strain-age cracking. More critically, these internal defects exhibit concealment, stochastic distribution, and micro-scale characteristics, challenging current non-destructive testing (NDT) technologies in resolution, penetration depth, and applicability to complex internal geometries, thereby hindering comprehensive, high-precision defect detection and quantification. Minor fluctuations in process parameters (e.g., laser power, scan speed, layer thickness) can induce pores, cracks, or other internal flaws that remain concealed yet critical [127]. Consequently, achieving precise closed-loop control of the manufacturing process is paramount for ensuring component quality and reproducibility [124]. As evidenced in Figure 23, even for relatively mature AM superalloys, performance consistency remains challenging—uncertainties are amplified further in geometrically intricate composites [63,126].

#### 6.1.3. Certification and Standardization

The absence of tailored certification frameworks constitutes perhaps the most critical bottleneck for aerospace CAM adoption. Current qualification pathways—rooted in subtractive manufacturing paradigms like AS9100D’s linear “design-fabricate-inspect” approach—fail to accommodate AM’s integrated process–structure–property interdependencies [128]. Aeronautical certification requires CAM-specific standards for defect acceptance in fatigue-critical aircraft parts, which are absent in current frameworks like AS9100D. Three core gaps persist: First, existing material specifications (e.g., AMS 2750H for pyrometry) lack test methods for AM-specific anomalies like layer-wise defect formation or anisotropic properties in continuous fiber composites [129]. For instance, ASTM D3039’s assumption of uniform anisotropy renders it inadequate for evaluating 3D-printed CFRTPCs where interlaminar shear strength (2.81 MPa) can be ≤5% of in-plane tensile strength (147 MPa) [130]. Second, process qualification standards neglect AM’s digital thread—critical variables (e.g., laser power hysteresis, in situ thermal history) remain unstandardized, preventing reproducible part substantiation. NASA’s GRX-810 superalloy case (Figure 23) exemplifies this: ±5% laser power deviation alters oxide dispersion efficacy, directly impacting creep life at 1093 °C [63]. Third, NDE protocols lack resolution for internal defects characteristic of CAM, such as micron-scale interfacial debonding in MMCs or fiber misalignment in DIW-printed ceramics. Without accepted certification methodologies, such as digital twins that link process monitoring to structural performance, CAM components will remain confined to non-critical applications. This forces the industry to rely on costly, legacy qualification paradigms for primary structures [131,132].

#### 6.1.4. Economics

Ultimately, economic viability serves as the decisive criterion for widespread technological adoption. In the aerospace sector, AM incurs substantially higher application costs—particularly during initial investment and R&D phases—encompassing expensive equipment, specialized materials, and professional training expenditures. Moreover, the dedicated supply chain ecosystem remains nascent; limited availability of metal powder bed fusion service providers constrains cost-effective scaled production, while AM platform costs and productivity require further optimization [133]. Stringent safety and performance standards prolong certification and adoption timelines, resulting in protracted return-on-investment cycles. Although AM reduces material waste and simplifies assembly, most processes cannot compete with conventional mass production in throughput efficiency. Consequently, demonstrating comprehensive economic advantages over traditional manufacturing through application-specific life cycle cost (LCC) analysis constitutes the critical driver for industrial implementation.

### 6.2. Sustainability and Environmental Considerations for CAM

Although often considered a green technology, the sustainability of CAM requires a rigorous life cycle assessment (LCA) to provide a balanced perspective [134].

#### 6.2.1. Traditional vs. AM: Comparative Analysis

CAM technology’s most significant environmental advantage lies in its exceptionally high material utilization efficiency. Conventional manufacturing processes typically involve material removal operations—such as machining and grinding—that generate substantial waste streams. These byproducts not only squander valuable resources but also impose environmental burdens through handling and disposal requirements. Traditional methods further employ various chemicals including coolants, lubricants, and cleaning agents, whose usage and disposal potentially contribute to environmental contamination. In contrast, AM builds components through layer-wise material deposition, drastically minimizing waste generation. This efficient material usage substantially reduces waste output, thereby diminishing environmental impacts; concurrently, AM processes offer greater optimization potential for overall energy consumption compared to conventional manufacturing [135]. Furthermore, CAM-produced lightweight components directly reduce aircraft fuel consumption and carbon emissions during operational phases, delivering substantial environmental benefits throughout service life.

#### 6.2.2. Environmental Impact

However, during the manufacturing phase, numerous CAM processes—particularly powder bed techniques requiring sustained heating or high-energy beams (e.g., SLS, EBM)—exhibit substantially higher energy consumption per unit than conventional mass production methods [135]. Within aerospace composites, integrating AM with sustainable development is emerging as a pivotal force driving industry advancement. This synergy not only enhances production efficiency and reduces costs but also promotes resource conservation and environmental protection, aligning with green aviation initiatives. For instance, AM of high-performance lightweight polymers and composites enables fabrication of structural components with superior mechanical properties and radar-absorbing functional parts [102], advancing integrated structure–function solutions. Furthermore, AM applications in manned space programs—such as on-orbit fabrication of spacecraft replacement components [136] and manufacturing of large trusses impractical for ground-based production or launch—demonstrate its critical role in achieving sustainable development goals. Regarding material recycling, Boeing and COMAC collaborate on sustainable cabin materials like ramie fiber-reinforced polylactic acid (PLA) composites, offering lightweight, high-strength, and 100% biodegradable properties. Airbus partners have developed recyclable rudders from thermoplastic honeycomb sandwich composites [137], enhancing end-of-life aircraft component recovery rates. Recycling technologies for carbon CFRP [138], mitigate the environmental impacts of aerospace carbon waste, embodying material circularity. Chatzipanagiotou et al. developed a supercritical water decomposition technology, achieving a 90% carbon fiber recovery rate with >90% strength retention [139]. Concurrently, producing AM-specific high-quality spherical powders or specialized filaments remains energy-intensive, with non-negligible environmental footprints. Most polymer matrix materials (e.g., PEEK, epoxy resins) derive from fossil sources, carrying intrinsic carbon emissions throughout their production lifecycle.

#### 6.2.3. Sustainable Development Pathways

Consequently, enhancing CAM’s sustainability hinges on establishing a closed-loop circular economy model. Aligned with the UN’s 17 Sustainable Development Goals, AM technologies empower designers to achieve exceptional precision while reducing expenditures, accelerating aerospace adoption. Efficient manufacturing processes may curtail production and logistics timelines/costs, distributing significant economic benefits across the value chain. AM’s core strengths—producing lighter, more resilient, and stronger components while achieving near-zero waste in aerospace production [140]—enable environmentally sound material reuse within circular systems [141]. Through AM implementation, aerospace resource efficiency has markedly improved, with material consumption-to-flight efficiency ratios approaching 1:1 [142]. However, persistent drawbacks remain: many composite AM processes (e.g., SLS, FDM) require sustained heating, high-energy lasers, or UV sources, exhibiting substantially higher energy intensity per unit than conventional mass production, while post-processing also consumes considerable energy. Most thermoplastic composites rely heavily on petrochemical feedstocks, incurring high energy consumption and carbon emissions during production. Research must prioritize developing recyclable/degradable thermoplastics and novel thermosetting resin systems alongside lower-energy AM technologies. The ultimate objective entails transitioning from a linear “cradle-to-grave” economy to a regenerative “cradle-to-cradle” model with inherent recyclability—the essential pathway for CAM to achieve genuine sustainability.

## 7. Future Outlook and Strategic Research Directions

Future composite materials will be developed for improved performance, manufacturability, cost-effectiveness, and sustainability. As the use of carbon fiber composites expands, effective recycling technologies represent a critical research frontier. Concurrently, the evolution toward large-scale integral structures will continue, aiming to reduce assembly, enhance structural integrity, and improve flight efficiency.

### 7.1. Development of Multifunctional and Smart Composite Materials

Future research will focus on multifunctional and smart composites. Multifunctional composites integrate static capabilities (e.g., load-bearing, thermal management), whereas smart composites actively respond to external stimuli via embedded sensors or actuators. AM enables significant design freedom for both systems. At the material level, incorporating nanofillers (e.g., CNTs, graphene) can enhance electrical, thermal, and mechanical properties, as demonstrated by FDM-fabricated composites for electromagnetic shielding [143]. At the process level, multi-material AM enables graded distribution of heterogeneous materials and functional integration.

### 7.2. Advancements in Hybrid Manufacturing Techniques

Hybrid manufacturing innovations primarily manifest through synergistic process optimization. For instance, combining laser-directed energy deposition (LDED) with Shot Peening (SP) enables strategic SP parameter tuning to elevate compressive residual surface stress to 2.26 times tensile stress while increasing density by 8.83% [144]. Future advancements should explore synergistic mechanisms between nano-reinforcements (e.g., graphene, SiC nanowires) and continuous fibers to develop next-generation high-strength lightweight composites.

### 7.3. AI-Driven Process Optimization and In Situ Monitoring

For WAAM dynamic thermo-mechanical behaviors, Min et al. [145] developed a deep neural network (DNN) model that achieves high-precision prediction of bead geometry (width/height) by analyzing wire feed rate and travel speed, providing theoretical guidance for process optimization. Regarding FDM of CFRP, researchers established an artificial neural network (ANN)-based fiber orientation prediction model, enhancing alignment accuracy through analysis of nozzle temperature, layer thickness, and print speed interactions [146]. In optical monitoring, integrated multi-spectrometer and high-speed camera systems synchronously capture high-fidelity melt pool temperature fields and morphological evolution, enabling millisecond-level tracking via feature fusion algorithms [147]. Future research priorities should focus on developing ultra-high-resolution, high-speed imaging and distributed sensing technologies to capture transient micro-defects; establishing in situ multi-physics monitoring models coupling thermal–mechanical–chemical–acoustic fields to deepen understanding of defect formation mechanisms; and enhancing AI algorithms’ adaptability and few-shot defect recognition accuracy to achieve real-time decision-making and dynamic process optimization.

### 7.4. Sustainability and Recycling of Composite AM Materials

Amid escalating global focus on the circular economy and low-carbon manufacturing, reducing the environmental footprint of AM processes while achieving closed-loop material utilization has become a shared challenge for academia and industry. CFRP recycling represents a critical research frontier. Solvolysis techniques employing supercritical water or plasma-assisted resin decomposition achieve 90% fiber recovery with surface characteristics comparable to virgin fibers [139]. In closed-loop material design, thermoplastic polycarbonate/carbon fiber composites fabricated via two-stage hot pressing demonstrate enhanced energy absorption in impact testing while offering superior recyclability over thermosetting systems [148]. Advancing recycling technologies constitutes concrete action for the industry to fulfill social accountability and practice responsible research innovation. Proactively addressing recycling challenges amid growing public environmental consciousness significantly elevates aerospace’s environmental stewardship and social acceptance. Investment in recycling technology directly underpins the long-term competitiveness, environmental compliance, and social license of aerospace AM.

## 8. Conclusions

This comprehensive review delineates the recent advancements, application exemplars, and future trajectories of CAM technology within the aerospace sector. We systematically analyze the application-specific characteristics of diverse composite systems—including thermoplastics, thermosets, metal matrix, and ceramic matrix composites—in AM contexts through the pioneering MPDP framework. Detailed examination encompasses CAM-adapted processes such as FDM, SLS, DIW, and AFP, with aerospace implementation cases substantiating their technical viability. Concurrently, this work critically addresses prevailing challenges and emerging research frontiers, providing a structured reference for researchers and practitioners. The principal conclusions and priority future directions are synthesized as follows:

(1) Technological Integration and MPDP Synergy:

CAM synergizes high-performance materials with precision fabrication to deliver unprecedented structural freedom and functional integration. For aeronautical applications, CAM’s adoption hinges on co-optimizing TRL 6–7 technologies (e.g., continuous fiber AM) with digital twin-enabled certification protocols to bridge the transition from lab-scale innovation to airworthy production. Future work must co-optimize deposition rates (e.g., via multi-axis robotic VDD) with in situ quality monitoring (e.g., AI-driven IR thermography) to close the gap with AFP (TRL 9).

(2) Material Innovations and Certification Gaps:

High-temperature polyimides (Tg > 370 °C) and smart composites (e.g., self-healing microcapsules) show promise for extreme environments but lack standardized testing protocols. The priority is establishing CAM-specific certification frameworks (e.g., digital twin-enabled NDE for micron-scale defects) to accelerate adoption in primary structures.

(3) Design-Driven Performance Leaps:

TO and self-supporting designs (e.g., Airbus A380 bracket: 45% weight reduction) demonstrate AM’s transformative potential. Urgent focus: Embed process constraints (e.g., thermal stress distribution) upfront in DfAM tools to avoid 57× post-processing penalties (Table 3).

(4) Sustainability Imperatives:

CAM reduces material waste by 20–40% versus subtractive methods but suffers from energy-intensive processes (e.g., SLS). Strategic pathway: Develop closed-loop recycling for CFRPs (>90% strength retention) and low-energy AM (e.g., DIW) to align with EU Clean Aviation targets (≥30% recycled content).

(5) Smart Manufacturing Convergence:

Embedded sensors (e.g., CNT/PLA strain gauges) enable real-time health monitoring but face EMI and high-temperature attenuation (>300 °C). Key R&D: Hybrid processes (e.g., UAM-embedded FBG sensors) with signal standardization for predictive maintenance.

While this review aimed for comprehensiveness, the rapid evolution of CAM technologies inevitably means that there are some very recent developments. The depth of analysis for each specific material system and each AM process is necessarily constrained by the breadth required for a holistic ‘Material-Process-Design-Performance’ framework analysis. Deeper, process-specific or material-specific mechanistic discussions are beyond the scope of this critical review. The analysis relies heavily on published literature, which may introduce publication bias (e.g., underreporting of negative results or unsuccessful trials) and variations in reporting standards across different studies, making direct quantitative comparisons challenging. Discussions on highly innovative but less mature areas, such as smart composites with integrated sensing/actuation (Section 5.3.4) or large-scale AM for primary structures (Section 5.3.3), are inherently limited by the scarcity of long-term performance data, standardized testing results, and certified application records in the public domain. To transcend these constraints, future work must prioritize the following:

(1) AI-driven co-optimization of material formulations and process parameters (e.g., melt pool control for MMCs).

(2) For aircraft applications, establishing industry–academia consortia to develop shared databases for CAM-specific fatigue and damage tolerance data, accelerating FAA/EASA certification.

(3) Digital thread integration linking real-time process monitoring to structural performance prediction.

Through convergence of the MPDP pillars, CAM is poised to achieve scalable, intelligent, and sustainable industrialization—propelling transformative next-generation aerospace systems characterized by integrated lightweight structures, multifunctional capabilities, and enhanced operational efficiency.

## Figures and Tables

**Table 1:** Comparative analysis of key AM technologies. (Part 1: materials to key advantages.)

AM Technology	Materials	Resolution	Typical Aerospace Applications	Key Advantages
FDM	PEEK, PEKK, ULTEM, Nylon composites	100–300 μm [48]	Ducts, brackets, cabin prototypes, tooling	Low cost, material diversity, desktop scalability
SLS	Nylon (PA), PA-CF, PEEK	50–300 μm [49]	Piping, jigs, lightweight housings	Support-free, chemical resistance, complex geometries
LMD	Ti-6Al-4V, Inconel 718, Stainless steel	100–500 μm [50]	Component repair, functionally graded materials, large features	High deposition rate, repair capability, large scale
SLM	Ti-6Al-4V, AlSi10Mg, Inconel 718	50–100 μm [51]	Complex structures (brackets, nozzles), heat exchangers	High precision, excellent mechanical properties, complexity
EBM	Ti-6Al-4V, Inconel 718, Nb alloys	50–200 μm [52]	High-temp components (blades, combustors)	Low residual stress, high-temp materials, vacuum environment
DIW	Ceramic slurries, Conductive inks, Composites	10–200 μm [53] (Dependent on Nozzle Diameter)	Radomes, sensors, custom ceramics	Multi-material, fine features, functional materials
AFP	Thermoplastic/Thermoset prepregs (CF, GF, Kevlar)	Fiber Width [54,55] (3200–12700 μm)	Wing/fuselage skins, spars, inlet ducts	High efficiency (large parts), high-performance composites, automation

**Table 2:** Comparative analysis of key AM technologies. (Part 2: key limitations to TRL.)

AM Technology	Key Limitations	Typical Post-Processing	Design Considerations	TRL
FDM	Strong anisotropy, low strength/temperature, poor surface finish	Support removal, sanding	Supports for overhangs, wall thickness limits, layer visibility	7–8
SLS	Powder handling, porosity, limited high-temp materials	Powder removal, bead blasting, impregnation	Powder removal channels, minimum wall thickness, self-supporting angles	7
LMD	Low accuracy/roughness, large heat-affected zone	Machining, grinding, HIP (optional)	Path planning, thermal input control, distortion compensation	8
SLM	High cost, residual stress, size limits, post-processing	Support removal, HIP, heat treatment, finish machining	Support optimization, avoidance of large flat areas, thermal warping	9
EBM	High equipment cost, vacuum chamber, rough surface, size limits	Support removal, HIP (optional), wire EDM	Minimal supports, avoidance of sharp corners, thermal warping	8
DIW	Low strength, post-processing (sintering), slow speed	Debinding, sintering, machining	Extrusion path planning, overhang limits, drying shrinkage	5–6
AFP	Massive/costly equipment, limited to laminates, complex curvature design	Autoclave curing, trimming, NDT	Ply design (angles/sequence), layup accessibility, minimum bend radius	9

**Table 3:** Comparison of three design strategies [64].

		TO		TO +ULS		TO +FGLS	
		Bracket A	Bracket B	Bracket A	Bracket B	Bracket A	Bracket B
Support structure requirements	Absolute (mm^2^)	3975	5714	4688	6325	6438	6759
	Relative value	0.62	0.85	0.73	0.94	1	1
Processing effort	Processing time (s)	6	14	257	625	343	716
	Relative value	0.02	0.02	0.75	0.87	1	1
Weight saving (%)	Absolute value	8.47	34.59	20.62	40.01	24.31	52.51
	Relative value	0.35	0.66	0.85	0.76	1	1

## Data Availability

No new data were created or analyzed in this study. Data sharing is not applicable to this article.

## References

[1] Zhang X., Chen Y., Hu J. (2018). Recent advances in the development of aerospace materials. Prog. Aerosp. Sci..

[2] Tiwary A., Kumar R., Chohan J.S. (2022). A review on characteristics of composite and advanced materials used for aerospace applications. Mater. Today Proc..

[3] Al-Mamun A., Hossain M., Iqbal M. (2024). Recent developments in the synthesis of composite materials for aerospace: Case study. Mater. Sci. Eng..

[4] Abidin N.M., Sultan M.T., Hua L.S., Basri A.A., Md Shah A.U., Safri S.N. (2019). A brief review of computational analysis and experimental models of composite materials for aerospace applications. J. Reinf. Plast. Compos..

[5] Shetu M. (2024). A review of nondestructive testing methods for aerospace composite materials. J. Comput. Mech. Manag..

[6] Saleh Alghamdi S., John S., Roy Choudhury N., Dutta N.K. (2021). Additive manufacturing of polymer materials: Progress, promise and challenges. Polymers.

[7] Sioutis I., Tserpes K. (2023). A literature review on crack arrest features for composite materials and composite joints with a focus on aerospace applications. Aerospace.

[8] Oliveira T.L.L., Hadded M., Mimouni S., Schaan R.B. (2025). The Role of Non-Destructive Testing of Composite Materials for Aerospace Applications. NDT.

[9] Montanari R., Palombi A., Richetta M., Varone A. (2023). Additive manufacturing of aluminum alloys for aeronautic applications: Advantages and problems. Metals.

[10] Peng J., Liu S., Wang D., Xu A., Huang X., Ma T., Wang J., Li H. (2024). Design and Optimization of Thin-Walled Main Support Structure for Space Camera Based on Additive Manufacturing. Micromachines.

[11] Slayton R., Spinardi G. (2016). Radical innovation in scaling up: Boeing’s Dreamliner and the challenge of socio-technical transitions. Technovation.

[12] Kontiza A., Kartsonakis I.A. (2024). Smart composite materials with self-healing properties: A review on design and applications. Polymers.

[13] AL-Oqla F.M., Hayajneh M.T., Nawafleh N. (2023). Advanced synthetic and biobased composite materials in sustainable applications: A comprehensive review. Emergent Mater..

[14] Matsuzaki R., Ueda M., Namiki M., Jeong T.-K., Asahara H., Horiguchi K., Nakamura T., Todoroki A., Hirano Y. (2016). Three-dimensional printing of continuous-fiber composites by in-nozzle impregnation. Sci. Rep..

[15] Blakey-Milner B., Gradl P., Snedden G., Brooks M., Pitot J., Lopez E., Leary M., Berto F., Du Plessis A. (2021). Metal additive manufacturing in aerospace: A review. Mater. Des..

[16] Van De Steene W., Ragaert K., Cardon L. Optimisation of a continuous fibre reinforced additive manufacturing process. Proceedings of the 35th International Conference of the Polymer Processing Society (PPS-35).

[17] Shan Z., Song W., Fan C., Wang J. (2023). Development strategy for precision manufacturing of composite components facing 2035. Strateg. Study Chin. Acad. Eng..

[18] Zhu Q., Yu X., Yao P., Yue Y., Kang G. (2024). Study on optimization strategy for the composition transition gradient in SS 316L/Inconel 625 functionally graded materials. Materials.

[19] Farsadi T., Rahmanian M., Kurtaran H. (2022). Nonlinear stability of multilayered graphene platelet-reinforced functionally graded wing-like plates. Acta Mech..

[20] Chao Q., Cruz V., Thomas S., Birbilis N., Collins P., Taylor A., Hodgson P.D., Fabijanic D. (2017). On the enhanced corrosion resistance of a selective laser melted austenitic stainless steel. Scr. Mater..

[21] Kunc V., Kishore V., Chen X., Ajinjeru C., Duty C., Hassen A.A. (2016). High Performance Poly (Etherketoneketone)(PEKK) Composite Parts Fabricated Using Big Area Additive Manufacturing (BAAM) Processes.

[22] Wang P., Zou B., Xiao H., Ding S., Huang C. (2019). Effects of printing parameters of fused deposition modeling on mechanical properties, surface quality, and microstructure of PEEK. J. Mater. Process. Technol..

[23] Majko J., Vaško M., Handrik M., Sága M. (2022). Tensile properties of additively manufactured thermoplastic composites reinforced with chopped carbon fibre. Materials.

[24] Penumakala P.K., Santo J., Thomas A. (2020). A critical review on the fused deposition modeling of thermoplastic polymer composites. Compos. Part B Eng..

[25] Guo S.Z., Qiu K., Meng F., Park S.H., McAlpine M.C. (2017). 3D printed stretchable tactile sensors. Adv. Mater..

[26] Ligon S.C., Liska R., Stampfl J., Gurr M., Mülhaupt R. (2017). Polymers for 3D printing and customized additive manufacturing. Chem. Rev..

[27] Tekoglu E., Bae J.-S., Kim H.-A., Lim K.-H., Liu J., Doležal T.D., Kim S.Y., Alrizqi M.A., Penn A., Chen W. (2024). Superior high-temperature mechanical properties and microstructural features of LPBF-printed In625-based metal matrix composites. Mater. Today.

[28] Wong C.S., Pramanik A., Basak A. (2018). Residual stress generation in metal matrix composites after cooling. Mater. Sci. Technol..

[29] Sciti D., Zoli L., Reimer T., Vinci A., Galizia P. (2022). A systematic approach for horizontal and vertical scale up of sintered Ultra-High Temperature Ceramic Matrix Composites for aerospace—Advances and perspectives. Compos. Part B Eng..

[30] O’Masta M.R., Stonkevitch E., Porter K.A., Bui P.P., Eckel Z.C., Schaedler T.A. (2020). Additive manufacturing of polymer-derived ceramic matrix composites. J. Am. Ceram. Soc..

[31] Patel R., Desai C., Kushwah S., Mangrola M. (2022). A review article on FDM process parameters in 3D printing for composite materials. Mater. Today Proc..

[32] Zhang H., Hui J., Lv J., Lee C.-H., Yan Z., Wang J.J., Guo L., Xu Z. (2023). A novel method to combine fused deposition modelling and inkjet printing in manufacturing multifunctional parts for aerospace application. J. Mater. Res. Technol..

[33] Byberg K.I., Gebisa A.W., Lemu H.G. (2018). Mechanical properties of ULTEM 9085 material processed by fused deposition modeling. Polym. Test..

[34] Kumar M.B., Sathiya P., Varatharajulu M. (2021). Selective laser sintering. Advances in Additive Manufacturing Processes.

[35] Wang Q., Gao M., Li L., Ma Z., Liu C. (2021). Emergy-based environmental impact evaluation and modeling of selective laser melting. Int. J. Adv. Manuf. Technol..

[36] Song S., Gao Z., Lu B., Bao C., Zheng B., Wang L. (2020). Performance optimization of complicated structural SiC/Si composite ceramics prepared by selective laser sintering. Ceram. Int..

[37] Daneshmand S., Aghanajafi C., Shahverdi H. (2012). Investigation of rapid manufacturing technology with ABS material for wind tunnel models fabrication. J. Polym. Eng..

[38] Liu W., Ren X., Li N., Gao C., Xiong H. (2021). Rapid directionally solidified microstructure characteristic and fracture behaviour of laser melting deposited Nb–Si–Ti alloy. Prog. Nat. Sci. Mater. Int..

[39] Wu Y., Sun K., Yu S., Zuo L. (2021). Modeling the selective laser melting-based additive manufacturing of thermoelectric powders. Addit. Manuf..

[40] Yakout M., Elbestawi M., Veldhuis S.C. (2018). On the characterization of stainless steel 316L parts produced by selective laser melting. Int. J. Adv. Manuf. Technol..

[41] Zafar M.Q., Wu C.C., Zhao H., Wang J., Hu X. (2020). Finite element framework for electron beam melting process simulation. Int. J. Adv. Manuf. Technol..

[42] Terrazas C.A., Gaytan S.M., Rodriguez E., Espalin D., Murr L.E., Medina F., Wicker R.B. (2014). Multi-material metallic structure fabrication using electron beam melting. Int. J. Adv. Manuf. Technol..

[43] Cao J., Gharghouri M.A., Nash P. (2016). Finite-element analysis and experimental validation of thermal residual stress and distortion in electron beam additive manufactured Ti-6Al-4V build plates. J. Mater. Process. Technol..

[44] Fernandez F., Compel W.S., Lewicki J.P., Tortorelli D.A. (2019). Optimal design of fiber reinforced composite structures and their direct ink write fabrication. Comput. Methods Appl. Mech. Eng..

[45] Harik R. (2020). ADVANCING COMPOSITES MANUFACTURING: neXt Automated Fiber Placement: Advancing Composites Manufacturing Towards a New Paradigm. SAMPE J..

[46] Sun S., Han Z., Zhang J., Jin H., Wang Y. (2021). Multiscale collaborative process optimization method for automated fiber placement. Compos. Struct..

[47] Nguyen M.H., Vijayachandran A.A., Davidson P., Call D., Lee D., Waas A.M. (2019). Effect of automated fiber placement (AFP) manufacturing signature on mechanical performance of composite structures. Compos. Struct..

[48] Al-Tamimi A.A., Pandi A., Kadri E. (2023). Investigation and Prediction of Tensile, Flexural, and Compressive Properties of Tough PLA Material Using Definitive Screening Design. Polymers.

[49] Franco A., Lanzetta M., Romoli L. (2010). Experimental analysis of selective laser sintering of polyamide powders: An energy perspective. J. Clean. Prod..

[50] Wang Q., Zhang J., Zhu Q., Cao Y. (2024). Closed-Loop Control of Melt Pool Temperature during Laser Metal Deposition. Sensors.

[51] Matsui M., Komatsu T., Sakamoto S., Itoh Y., Sakai K., Shizuka H., Miura H. (2018). Fabrication of precision and complex-shaped parts using a developed SLM machine. Funtai Oyobi Fummatsu Yakin/J. Jpn. Soc. Powder Powder Metall..

[52] Tebianian M., Aghaie S., Razavi Jafari N.S., Elmi Hosseini S.R., Pereira A.B., Fernandes F.A.O., Farbakhti M., Chen C., Huo Y. (2023). A Review of the Metal Additive Manufacturing Processes. Materials.

[53] Siqueira G., Kokkinis D., Libanori R., Hausmann M.K., Gladman A.S., Neels A., Tingaut P., Zimmermann T., Lewis J.A., Studart A.R. (2017). Cellulose Nanocrystal Inks for 3D Printing of Textured Cellular Architectures. Adv. Funct. Mater..

[54] Rajainmaki H., Foussat A., Rodriguez J., Evans D., Fanthome J., Losasso M., Diaz V. The ITER pre-compression rings—A first in cryogenic composite technology. Proceedings of the 2013 Joint Cryogenic Engineering and International Cryogenic Materials Conferences (ICMC 2013).

[55] Wei Y., Liu J., Zhu D., He C., Luo J., Xu T., Li B., Li X. Buckling stability of variable stiffness composite cylinder shell based on automated fiber placement technology. Proceedings of the Chinese Materials Conference 2022–2023.

[56] Lindwall A., Dordlofva C., Öhrwall Rönnbäck A., Törlind P. (2022). Innovation in a box: Exploring creativity in design for additive manufacturing in a regulated industry. J. Eng. Des..

[57] Haleem A., Javaid M. (2020). 3D printed medical parts with different materials using additive manufacturing. Clin. Epidemiol. Glob. Health.

[58] Dong G., Tang Y., Li D., Zhao Y.F. (2020). Design and optimization of solid lattice hybrid structures fabricated by additive manufacturing. Addit. Manuf..

[59] Henson M., Barker D., Eby B., Weber C., Wang B., Koenig J. Advanced tools for rapid development of reduced complexity composite structures. Proceedings of the 43rd AIAA/ASME/ASCE/AHS/ASC Structures, Structural Dynamics, and Materials Conference.

[60] Snyder J.C., Thole K.A. (2020). Effect of additive manufacturing process parameters on turbine cooling. J. Turbomach..

[61] Shi G., Guan C., Quan D., Wu D., Tang L., Gao T. (2020). An aerospace bracket designed by thermo-elastic topology optimization and manufactured by additive manufacturing. Chin. J. Aeronaut..

[62] Ranjan R., Samant R., Anand S. (2017). Integration of design for manufacturing methods with topology optimization in additive manufacturing. J. Manuf. Sci. Eng..

[63] Gradl P., Mireles O.R., Katsarelis C., Smith T.M., Sowards J., Park A., Chen P., Tinker D.C., Protz C., Teasley T. (2023). Advancement of extreme environment additively manufactured alloys for next generation space propulsion applications. Acta Astronaut..

[64] Gairola S., Jayaganthan R. (2025). Lattice infill strategies for topology optimisation towards achieving lightweight designs for additive manufacturing: Structural integrity, and manufacturing consideration. J. Manuf. Process..

[65] Fu Y.-F., Rolfe B., Chiu L.N., Wang Y., Huang X., Ghabraie K. (2019). Design and experimental validation of self-supporting topologies for additive manufacturing. Virtual Phys. Prototyp..

[66] Wang D., Yang Y., Yi Z., Su X. (2013). Research on the fabricating quality optimization of the overhanging surface in SLM process. Int. J. Adv. Manuf. Technol..

[67] Mertens R., Clijsters S., Kempen K., Kruth J.-P. (2014). Optimization of scan strategies in selective laser melting of aluminum parts with downfacing areas. J. Manuf. Sci. Eng..

[68] Langelaar M. (2016). Topology optimization of 3D self-supporting structures for additive manufacturing. Addit. Manuf..

[69] Mezzadri F., Bouriakov V., Qian X. (2018). Topology optimization of self-supporting support structures for additive manufacturing. Addit. Manuf..

[70] Jia H., Duan S., Zhang Z., Yen C.-C., Feng Lu W., Lei H. (2024). Homogenization-based topology optimization for self-supporting additive-manufactured lattice-infilled structure. Mater. Des..

[71] Zhang B., Goel A., Ghalsasi O., Anand S. (2019). CAD-based design and pre-processing tools for additive manufacturing. J. Manuf. Syst..

[72] Chen F., Mac G., Gupta N. (2017). Security features embedded in computer aided design (CAD) solid models for additive manufacturing. Mater. Des..

[73] Gu D., Shi X., Poprawe R., Bourell D.L., Setchi R., Zhu J. (2021). Material-structure-performance integrated laser-metal additive manufacturing. Science.

[74] Shamsujjoha M., Agnew S.R., Fitz-Gerald J.M., Moore W.R., Newman T.A. (2018). High strength and ductility of additively manufactured 316L stainless steel explained. Metall. Mater. Trans. A.

[75] Liu L., Ding Q., Zhong Y., Zou J., Wu J., Chiu Y.-L., Li J., Zhang Z., Yu Q., Shen Z. (2018). Dislocation network in additive manufactured steel breaks strength–ductility trade-off. Mater. Today.

[76] Park J.M., Choe J., Kim J.G., Bae J.W., Moon J., Yang S., Kim K.T., Yu J.-H., Kim H.S. (2020). Superior tensile properties of 1% C-CoCrFeMnNi high-entropy alloy additively manufactured by selective laser melting. Mater. Res. Lett..

[77] Fujieda T., Chen M., Shiratori H., Kuwabara K., Yamanaka K., Koizumi Y., Chiba A., Watanabe S. (2019). Mechanical and corrosion properties of CoCrFeNiTi-based high-entropy alloy additive manufactured using selective laser melting. Addit. Manuf..

[78] Laleh M., Hughes A.E., Xu W., Cizek P., Tan M.Y. (2020). Unanticipated drastic decline in pitting corrosion resistance of additively manufactured 316L stainless steel after high-temperature post-processing. Corros. Sci..

[79] Yakout M., Cadamuro A., Elbestawi M., Veldhuis S.C. (2017). The selection of process parameters in additive manufacturing for aerospace alloys. Int. J. Adv. Manuf. Technol..

[80] Jiang Z., Zeng C., Chang Z., Li Z., Zhao Y., Cong B. (2025). Synergistic optimization of efficiency-microstructure-performance in wire-arc additive manufacturing of AZ31 magnesium alloy. J. Magnes. Alloys.

[81] Ma C., Gu D., Lin K., Dai D., Xia M., Yang J., Wang H. (2019). Selective laser melting additive manufacturing of cancer pagurus’s claw inspired bionic structures with high strength and toughness. Appl. Surf. Sci..

[82] Jin Y.-a., He Y., Fu J.-z., Gan W.-f., Lin Z.-w. (2014). Optimization of tool-path generation for material extrusion-based additive manufacturing technology. Addit. Manuf..

[83] Jiang J., Xu X., Stringer J. (2019). Optimization of process planning for reducing material waste in extrusion based additive manufacturing. Robot. Comput.-Integr. Manuf..

[84] Łakomy M., Kluczyński J., Sarzyński B., Jasik K., Szachogłuchowicz I., Łuszczek J. (2024). Bending Strength of Continuous Fiber-Reinforced (CFR) Polyamide-Based Composite Additively Manufactured through Material Extrusion. Materials.

[85] Du Z., Deng K., Nie K., Wang C., Xu C., Shi Q. (2023). High-modulus laminated SiC/AZ91 material with adjustable microstructure and mechanical properties based on the adjustment of the densities of the ceramic layers. Materials.

[86] Lin T.-C., Cao C., Sokoluk M., Jiang L., Wang X., Schoenung J.M., Lavernia E.J., Li X. (2019). Aluminum with dispersed nanoparticles by laser additive manufacturing. Nat. Commun..

[87] Zhao M., Liu F., Zhou H., Zhang T., Zhang D.Z., Fu G. (2023). Effect of the direction of the gradient on the mechanical properties and energy absorption of additive manufactured Ti-6Al-4 V functionally graded lattice structures. J. Alloys Compd..

[88] Jagannadham K. (2012). Thermal conductivity of copper-graphene composite films synthesized by electrochemical deposition with exfoliated graphene platelets. Metall. Mater. Trans. B.

[89] Omairey S.L., Sampethai S., Hans L., Worrall C., Lewis S., Negro D., Sattar T., Ferrera E., Blanco E., Wighton J. (2021). Development of innovative automated solutions for the assembly of multifunctional thermoplastic composite fuselage. Int. J. Adv. Manuf. Technol..

[90] Fasel U., Keidel D., Baumann L., Cavolina G., Eichenhofer M., Ermanni P. (2020). Composite additive manufacturing of morphing aerospace structures. Manuf. Lett..

[91] Kobenko S., Dejus D., Jātnieks J., Pazars D., Glaskova-Kuzmina T. (2022). Structural integrity of the aircraft interior spare parts produced by additive manufacturing. Polymers.

[92] Acanfora V., Corvino C., Saputo S., Sellitto A., Riccio A. (2021). Application of an additive manufactured hybrid metal/composite shock absorber panel to a military seat ejection system. Appl. Sci..

[93] Song X., He W., Ihara T. (2023). A novel approach for dry cutting inconel 718 in a more sustainable and low-cost way by actively and purposely utilizing the built-up layer. Micromachines.

[94] Keller A.R., Bendana F.A., Phong V.C., Spearrin R.M. (2024). Additively-manufactured shear tri-coaxial rocket injector mixing and combustion characteristics. Aerosp. Sci. Technol..

[95] Kazemi M.E., Medeau V., Chen Y., Xu Z., Petrinic N., Greenhalgh E., Robinson P., Finlayson J., Pinho S.T. (2024). Ballistic performance of bio-inspired hybrid interleaved composite structures suitable for aerospace applications. Compos. Part A Appl. Sci. Manuf..

[96] Wilhelmsson D., Rikemanson D., Bru T., Asp L.E. (2020). Compressive strength assessment of a CFRP aero-engine component—An approach based on measured fibre misalignment angles. Compos. Struct..

[97] Souza A., Gonçalves P.T., Afonso F., Lau F., Rocha N., Suleman A. (2024). On the multidisciplinary design of a hybrid rocket launcher with a composite overwrapped pressure vessel. J. Compos. Sci..

[98] Tomlin M., Meyer J. Topology optimization of an additive layer manufactured (ALM) aerospace part. Proceedings of the 7th Altair CAE technology conference.

[99] Meng L., Zhang W., Quan D., Shi G., Tang L., Hou Y., Breitkopf P., Zhu J., Gao T. (2020). From topology optimization design to additive manufacturing: Today’s success and tomorrow’s roadmap. Arch. Comput. Methods Eng..

[100] Zuo Z., Gong J., Huang Y., Zhan Y., Gong M., Zhang L. (2019). Experimental research on transition from scale 3D printing to full-size printing in construction. Constr. Build. Mater..

[101] Xiong J., Lei Y., Chen H., Zhang G. (2017). Fabrication of inclined thin-walled parts in multi-layer single-pass GMAW-based additive manufacturing with flat position deposition. J. Mater. Process. Technol..

[102] Zhang J., Fan Z., Li B., Ren D., Xu M. (2024). Study on structure–function integrated polymer-based microwave-absorption composites. Polymers.

[103] Min S., Tan Y., Zhang F., Tu Y. (2023). 3D printing process for continuous fibre reinforced composites with variable deposition direction. Adv. Mech. Eng..

[104] Xie F., Chen L., Li Z., Tang K. (2020). Path smoothing and feed rate planning for robotic curved layer additive manufacturing. Robot. Comput.-Integr. Manuf..

[105] Fang G., Zhang T., Huang Y., Zhang Z., Masania K., Wang C.C.L. (2024). Exceptional mechanical performance by spatial printing with continuous fiber: Curved slicing, toolpath generation and physical verification. Addit. Manuf..

[106] Akhoundi B., Behravesh A.H., Bagheri Saed A. (2019). Improving mechanical properties of continuous fiber-reinforced thermoplastic composites produced by FDM 3D printer. J. Reinf. Plast. Compos..

[107] Assadi M.D. (2021). High Speed AFP Processing of Thermoplastics. Int. SAMPE Tech. Conf..

[108] Heidari-Rarani M., Rafiee-Afarani M., Zahedi A.M. (2019). Mechanical characterization of FDM 3D printing of continuous carbon fiber reinforced PLA composites. Compos. Part B Eng..

[109] Chuang K.C., Gornet T.J., Schneidau K., Koerner H. Laser sintering of thermoset polyimide composites. Proceedings of the The Composites and Advanced Materials Expo.

[110] Gupta N., Srilekha G., Das K.K., Goel R., Mashkour M.S., Kumar M. (2023). Novel Manufacturing Techniques for Multifunctional Composites: Integration of Sensors and Actuators. E3S Web Conf..

[111] Gackowski B.M., Goh G.D., Sharma M., Idapalapati S. (2023). Additive manufacturing of nylon composites with embedded multi-material piezoresistive strain sensors for structural health monitoring. Compos. Part B Eng..

[112] Li P., Xia Y., Zhang C., Wang C., Liu Y., Fang H., Wang F. (2024). Mechanical and piezoresistive properties of multi-walled carbon nanotubes reinforced epoxy matrix composites for pipeline monitoring. J. Mater. Res. Technol..

[113] Tenney D.R., Davis J.G., Pipes R.B., Johnston N. NASA composite materials development: Lessons learned and future challenges. Proceedings of the NATO RTO AVT-164 Workshop on Support of Composite Systems.

[114] Gammelgaard L., Rasmussen P.A., Calleja M., Vettiger P., Boisen A. (2006). Microfabricated photoplastic cantilever with integrated photoplastic/carbon based piezoresistive strain sensor. Appl. Phys. Lett..

[115] Zhang C., Yu H., Sun D., Liu W. (2022). Ultrasonic additive manufacturing of metallic materials. Metals.

[116] Manns M., Raza S.M., Morez D., Schreiber F., Engel B. (2024). Embedding optical fiber with laser metal deposition. Prog. Addit. Manuf..

[117] Blaiszik B.J., Kramer S.L., Olugebefola S.C., Moore J.S., Sottos N.R., White S.R. (2010). Self-healing polymers and composites. Annu. Rev. Mater. Res..

[118] Kim H.-G., Hajra S., Oh D., Kim N., Kim H.J. (2021). Additive manufacturing of high-performance carbon-composites: An integrated multi-axis pressure and temperature monitoring sensor. Compos. Part B Eng..

[119] Luetkehoff B., Blum M., Schroeter M. Self-learning production control using algorithms of artificial intelligence. Proceedings of the Working Conference on Virtual Enterprises.

[120] Meng F., Olivetti E.A., Zhao Y., Chang J.C., Pickering S.J., McKechnie J. (2018). Comparing life cycle energy and global warming potential of carbon fiber composite recycling technologies and waste management options. ACS Sustain. Chem. Eng..

[121] Filippatos A., Markatos D., Tzortzinis G., Abhyankar K., Malefaki S., Gude M., Pantelakis S. (2024). Sustainability-driven design of aircraft composite components. Aerospace.

[122] Krinitcyn M., Kopytov G., Ryumin E. (2024). Additive Manufacturing of Ti3AlC2/TiC and Ti3AlC2/SiC Ceramics Using the Fused Granules Fabrication Technique. J. Manuf. Mater. Process..

[123] Yao Z., Xue S., Yang J., Zhang J. (2020). Inducing the effect of a Ga2O3 nano-particle on the CsF-RbF-AlF3 flux for brazing aluminum to carbon steels. Crystals.

[124] Khorasani M., Ghasemi A., Rolfe B., Gibson I. (2022). Additive manufacturing a powerful tool for the aerospace industry. Rapid Prototyp. J..

[125] Boyer R., Williams J., Wu X., Clark L. (2015). A realistic approach for qualification of PM applications in the aerospace industry. Titanium Powder Metallurgy.

[126] Daly M., Chihi M., Bouraoui C., Tarfaoui M. (2024). Advancing composite materials: Exploring thermomechanical properties of Aerosil/polycarbonate composites via additive manufacturing. J. Manuf. Process..

[127] Qian C., Zhang K., Zhu J., Liu Y., Liu Y., Liu J., Liu J., Yang Y., Wang H. (2024). Effect of processing parameters on the defects, microstructure, and property evaluation of Ti-6Al-4V titanium alloy processed by laser powder bed fusion. AIP Adv..

[128] SAE International (2016). AS9100D: Quality Management Systems—Requirements for Aviation, Space and Defense Organizations.

[129] SAE International (2024). AMS 2750H: Pyrometry.

[130] (2017). Standard Test Method for Tensile Properties of Polymer Matrix Composite Materials.

[131] Ghimire R., Liou F. (2021). Coupled flexural-electrical evaluation of additively manufactured multifunctional composites at ambient temperature. Appl. Sci..

[132] Dou H., Cheng Y., Ye W., Zhang D., Li J., Miao Z., Rudykh S. (2020). Effect of process parameters on tensile mechanical properties of 3D printing continuous carbon fiber-reinforced PLA composites. Materials.

[133] Baumers M., Dickens P., Tuck C., Hague R. (2016). The cost of additive manufacturing: Machine productivity, economies of scale and technology-push. Technol. Forecast. Soc. Change.

[134] Landi D., Zefinetti F.C., Spreafico C., Regazzoni D. (2022). Comparative life cycle assessment of two different manufacturing technologies: Laser additive manufacturing and traditional technique. Procedia CIRP.

[135] Ma Z., Gao M., Wang Q., Wang N., Li L., Liu C., Liu Z. (2021). Energy consumption distribution and optimization of additive manufacturing. Int. J. Adv. Manuf. Technol..

[136] Sears P.J., Ho K. (2018). Impact evaluation of in-space additive manufacturing and recycling technologies for on-orbit servicing. J. Spacecr. Rocket..

[137] Ma W., Elkin R. (2022). Sandwich Structural Composites: Theory and Practice.

[138] Li H., Englund K. (2017). Recycling of carbon fiber-reinforced thermoplastic composite wastes from the aerospace industry. J. Compos. Mater..

[139] Chatzipanagiotou K.-R., Antypa D., Petrakli F., Karatza A., Pikoń K., Bogacka M., Poranek N., Werle S., Amanatides E., Mataras D. (2023). Life Cycle Assessment of Composites Additive Manufacturing Using Recycled Materials. Sustainability.

[140] Verma V.K., Kamble S.S., Ganapathy L., Belhadi A., Gupta S. (2023). 3D Printing for sustainable food supply chains: Modelling the implementation barriers. Int. J. Logist. Res. Appl..

[141] Pinho A.C., Amaro A.M., Piedade A.P. (2020). 3D printing goes greener: Study of the properties of post-consumer recycled polymers for the manufacturing of engineering components. Waste Manag..

[142] Dutta B., Froes F.H. (2016). Additive Manufacturing of Titanium Alloys: State of the Art, Challenges and Opportunities.

[143] Vidakis N., Petousis M., Michailidis N., Mountakis N., Argyros A., Spiridaki M., Moutsopoulou A., Papadakis V., Charitidis C. (2023). High-density polyethylene/carbon black composites in material extrusion additive manufacturing: Conductivity, thermal, rheological, and mechanical responses. Polymers.

[144] Zhang X., Li D., Zhu W. (2020). Numerical modeling design for the hybrid additive manufacturing of laser directed energy deposition and shot peening forming Fe–Cr–Ni–B–Si alloy. Materials.

[145] So M.S., Mahdi M.M., Kim D.B., Shin J.-H. (2024). Prediction of Metal Additively Manufactured Bead Geometry Using Deep Neural Network. Sensors.

[146] Ghimire R., Raji A. (2024). Use of artificial intelligence in design, development, additive manufacturing, and certification of multifunctional composites for aircraft, drones, and spacecraft. Appl. Sci..

[147] May L., Werz M. (2024). Theoretical-numerical investigation of a new approach to reconstruct the temperature field in PBF-LB/M using multispectral process monitoring. J. Manuf. Mater. Process..

[148] Liu X., Yang B., Lu L., Wan Z., Tang Y. (2018). A thermoplastic multilayered carbon-fabric/polycarbonate laminate prepared by a two-step hot-press technique. Polymers.

